# Bayesian models for syndrome- and gene-specific probabilities of novel variant pathogenicity

**DOI:** 10.1186/s13073-014-0120-4

**Published:** 2015-01-28

**Authors:** Dace Ruklisa, James S Ware, Roddy Walsh, David J Balding, Stuart A Cook

**Affiliations:** UCL Genetics Institute, London, UK; NIHR Biomedical Research Unit in Cardiovascular Disease at Royal Brompton and Harefield NHS Foundation Trust and Imperial College, London, UK; National Heart and Lung Institute, Imperial College, London, UK; Current address: Department of Genetics and Department of Mathematics and Statistics, University of Melbourne, Melbourne, Australia; National Heart Centre, Singapore, Singapore; Duke-National University, Singapore, Singapore

## Abstract

**Background:**

With the advent of affordable and comprehensive sequencing technologies, access to molecular genetics for clinical diagnostics and research applications is increasing. However, variant interpretation remains challenging, and tools that close the gap between data generation and data interpretation are urgently required. Here we present a transferable approach to help address the limitations in variant annotation.

**Methods:**

We develop a network of Bayesian logistic regression models that integrate multiple lines of evidence to evaluate the probability that a rare variant is the cause of an individual’s disease. We present models for genes causing inherited cardiac conditions, though the framework is transferable to other genes and syndromes.

**Results:**

Our models report a probability of pathogenicity, rather than a categorisation into pathogenic or benign, which captures the inherent uncertainty of the prediction. We find that gene- and syndrome-specific models outperform genome-wide approaches, and that the integration of multiple lines of evidence performs better than individual predictors. The models are adaptable to incorporate new lines of evidence, and results can be combined with familial segregation data in a transparent and quantitative manner to further enhance predictions.

Though the probability scale is continuous, and innately interpretable, performance summaries based on thresholds are useful for comparisons. Using a threshold probability of pathogenicity of 0.9, we obtain a positive predictive value of 0.999 and sensitivity of 0.76 for the classification of variants known to cause long QT syndrome over the three most important genes, which represents sufficient accuracy to inform clinical decision-making. A web tool APPRAISE [http://www.cardiodb.org/APPRAISE] provides access to these models and predictions.

**Conclusions:**

Our Bayesian framework provides a transparent, flexible and robust framework for the analysis and interpretation of rare genetic variants. Models tailored to specific genes outperform genome-wide approaches, and can be sufficiently accurate to inform clinical decision-making.

**Electronic supplementary material:**

The online version of this article (doi:10.1186/s13073-014-0120-4) contains supplementary material, which is available to authorized users.

## Background

With ongoing technology developments, DNA sequencing is becoming increasingly feasible for a range of clinical conditions [1–4]. However, many healthy individuals carry rare variants in disease-associated genes, and our ability to interpret novel genetic findings struggles to keep pace with our ability to generate genetic data [5–8]. Thus, distinguishing genetic variants that cause disease from rare but benign variants is one of the principal challenges in contemporary clinical genetics.

To determine whether a novel variant found in a patient is likely to be pathogenic, well-powered segregation analysis or functional biochemical characterisation could be performed [9,10]. These are often impractical due to cost and time constraints, or a lack of phenotypically characterised family members for segregation studies. An alternative is to integrate evidence from various sources to make accurate predictions about the consequences of the observed variant, using approaches such as decision trees or more informal guidelines [9–12].

Here we develop a robust and transparent approach to predicting whether or not a novel protein-altering variant is the principal cause of a patient’s cardiac disease. We consider long QT syndrome (LQTS [MIM 192500]), Brugada syndrome (BrS [MIM 601144]) and hypertrophic cardiomyopathy (HCM [MIM 192600]). Our approach integrates sources of evidence including allele frequency, amino acid conservation, predictors based on physicochemical properties, and gene- and domain-specific effects, with odds ratios associated with each source of evidence estimated from training data specific to each syndrome. We specify syndrome- and gene-specific models because we expect them to be more accurate than genome-wide tools, as has been shown previously [12,13]. Differences in parameter values across genes or domains may be driven, for example, by whether the mechanism of pathogenesis involves gain or loss of function.

We require our framework to be transparent in its assumptions, expandable to include new kinds of evidence, and to report a probability of pathogenicity, rather than simply classifying the variant as benign or pathogenic for a syndrome. These requirements are met using Bayesian inference, in which the posterior odds of pathogenicity is computed as:

(1) $$ \text{Posterior odds} = \text{Prior odds} \times \text{Likelihood ratio}  $$

Recall that odds is related to probability (*P*) via Odds = *P*/(1−*P*), and that the likelihood ratio (LR) is given by:

(2) $$ {\fontsize{8}{12}\begin{aligned} \text{LR} = \frac{\text{Probability of observing the\! evidence if the variant is pathogenic}}{\text{Probability of observing the evidence if the variant is benign}} \end{aligned}}  $$

A simple approach to integrating multiple sources of evidence, called naïve Bayes, sequentially applies Equation (1) for each piece of evidence, therefore assuming independence [14]. This often works well when the predictor variables are not too correlated, but our predictors are often highly correlated and so joint modelling of all available predictor variables is required.

Bayesian frameworks have been previously used to integrate evidence for the interpretation of genetic variation, particularly in the assessment of variants that may cause familial cancer syndromes (discussed in [10,15]). Here, naïve Bayesian frameworks have been applied to combine sources of evidence for variant pathogenicity [16,17], and also to account for experimental variability in an *in vitro* assay used to validate *BRCA1* variant effects [18]. Recently Campbell *et al.* employed a similar framework to prioritise genes coincident with copy number variants for a likely role in the pathogenesis of epilepsy [19]. Here the authors were classifying genes, not variants, but there are parallels with the challenge we address. They derived empirical likelihood ratios for a number of predictors, then normalised and averaged them to yield a composite metric. Though the integration of individual predictors into the composite metric was not Bayesian, an explicit Bayesian framework was subsequently invoked to combine this pathogenicity score with information on background population rates of genetic variation in each gene. We use equivalent frequency data in our model to derive gene-level prior probabilities, which will be modified according to variant-level evidence to produce variant-specific predictions.

We also move from a naïve Bayesian approach to the development of a network of Bayesian logistic regression models, in which the probability that a variant is pathogenic is modelled in terms of a linear combination of predictor variables. For each syndrome, we develop a distinct linear predictor for each of three categories of coding variants: missense substitutions, inframe indels (insertions and deletions) and radical variants (nonsense substitutions, disruptors of consensus splice sites and frameshift indels). To fit the models, we use training sets of variants in known cardiac disease genes, which are identified as pathogenic or benign according to a rigorous definition detailed below.

## Methods

Ethical approval was not required, as this study employed reanalyses of published data in the public domain.

For each syndrome (LQTS, BrS and HCM), we identified a set of genes known to harbour pathogenic variants for that syndrome and, for each gene, derived prior odds that a rare variant is pathogenic. Next, we identified relevant predictors and used them to construct Bayesian logistic regression models, trained using a set of well-characterised rare variants. We now describe these steps in more detail.

### Prior odds of pathogenicity

The probability that an observed variant is pathogenic depends on the gene in which it is located and the syndrome of the patient. In a patient with LQTS for example, a variant in *KCNQ1* (a gene implicated in a large proportion of LQTS cases) has a higher probability of pathogenicity than a variant in *KCNJ5* (which has only been implicated in a handful of cases). Similarly, prior evidence indicates that a variant in the gene *MYH7* has a substantial probability to be pathogenic in an HCM patient, but a low probability of pathogenicity for LQTS.

The prior odds of pathogenicity for a rare variant in gene G, found in an affected individual, might be assumed to be:

(3) $$ {\fontsize{8.1}{12}\begin{aligned} \text{Prior odds of pathogenicity} = \frac{\text{Burden of pathogenic variants in cases}}{\text{Burden of benign rare variants in cases}} \end{aligned}}  $$

where burden means the proportion of individuals who have a variant in G of the type stated. Because the pathogenic/benign status of variants is often not known with certainty, we cannot directly estimate either the numerator or denominator of Equation (3). However, it is reasonable to assume that the burden of benign rare variants in cases is equal to the burden of rare variants in controls, which we estimate from population sequence data generated by the Exome Sequencing Project [20] (Table 1). Therefore, we could replace the right-hand side of Equation (3) with:

(4) $$  {\fontsize{8.2}{12}\begin{aligned} \!\frac{\text{Burden of rare variants in cases} - \text{Burden of rare variants in controls}}{\text{Burden of rare variants in controls}} \end{aligned}}  $$

**Table 1:** **Estimated burden of rare variants in cases and controls, for the calculation of prior odds of pathogenicity**

		**Non-radical variants**			**Radical variants**			
**Gene**	**Syndrome**	**Burden in**	**Burden in**	**Prior**	**Burden in**	**Burden in**	**Prior**	**Case burden in literature**
		**case series**	**controls**	**odds**	**case series**	**controls**	**odds**	**(combined radical and**
								**non-radical variants)**
*KCNQ1*	LQTS	0.1784	0.0039	45	0.0384	0.0002	191	0.421
*KCNH2*	LQTS	0.1256	0.0048	25	0.0392	0	195	0.388
*SCN5A*	LQTS	0.0584	0.0112	4	0.0028	0.0002	13	0.090
*KCNE1*	LQTS	0.0124	0.0014	8	0.0020	0	9	0.010
*KCNE2*	LQTS	0.0052	0.0011	4	0.0004	0.0002	1	0.010
*KCNJ2*	LQTS		0.0026	0.92		0	0.2	0.010
*ANK2*	LQTS		0.0386	0.2		0	0.2	0.010
*CACNA1C*	LQTS		0.0112	0.2		0	0.2	0.010
*CAV3*	LQTS		0.0011	4		0.0002	0.2	0.010
*SCN4B*	LQTS		0.0017	0.2		0.0006	0.2	0.001
*AKAP9*	LQTS		0.0393	0.2		0.0005	0.2	0.001
*SNTA1*	LQTS		0.0043	0.2		0.0003	0.2	0.001
*KCNJ5*	LQTS		0.0045	0.2		0	0.2	0.001
*MYBPC3*	HCM			37.5			37.5	0.375
*MYH7*	HCM			25			25	0.250
*TNNT2*	HCM			6			6	0.060
*TNNI3*	HCM			6			6	0.060
*SCN5A*	BrS			30			30	0.300
*CACNA1C*	BrS			1			1	0.010
*CACNA2D1*	BrS			1			1	0.010
*CACNB2*	BrS			1			1	0.010
*GPD1L*	BrS			1			1	0.010
*KCND3*	BrS			1			1	0.010
*KCNE3*	BrS			1			1	0.010
*SCN1B*	BrS			1			1	0.010
*SCN3B*	BrS			1			1	0.010

The estimated burden of rare variants in genes for LQTS, HCM and BrS in cases and ESP controls, where rare corresponds to an allele frequency <0.0005. Burdens are given as proportions (range 0-1). Values are presented for radical (truncating) variants and for inframe indels and missense substitutions combined (non-radical variants). For all genes, an estimate of the burden in cases is derived from the literature. For five LQTS genes, the burden in cases is estimated both from literature reports and from a prospective case series; the latter are used in our model where available. Otherwise half the literature-based estimate is used for LQTS genes (see text for discussion). Zero values of control frequencies are replaced by 0.0002 to compute ratios. For HCM and BrS the burden in cases is taken from the literature, and the burden in controls is fixed at 0.01.

The burden of rare variants in cases is estimated primarily from literature reports of the yields of diagnostic genetic testing for each gene and syndrome [21–24], as detailed in Additional file 1: Table S4 and summarised in Table 1.

In practice (Table 1), we made some adjustments to Equation (4). If any estimated burden of rare variants in the controls was zero, it was replaced by the minimum non-zero frequency (0.0002). This was done under the assumption that the minimum frequency observed corresponds to a variant count of 1. An alternative approach would be to transform frequencies with (*x*+1)/*n* where *x* is the count of variants and *n* is the number of individuals considered, but this information is not uniformly available. When the prior odds obtained from Equation (4) were smaller than 0.2, they were replaced by 0.2. Such a truncation was performed because estimation of the burden of rare variants in cases gets less precise in the lower range of values, and random fluctuations in estimated control frequencies have more impact upon the prior odds within this range of rare variant frequencies in cases. The threshold for truncation of prior odds was set to 0.2, following simulation experiments demonstrating that predictions were equally accurate when the prior odds were set at either 1 or 0.2.

We employed an alternative approach to calculating prior odds for five LQTS genes (*KCNQ1*, *KCNH2*, *SCN5A*, *KCNE1* and *KCNE2*) that is not based on the literature reports given in Additional file 1: Table S4. We obtained independent burden estimates from a published prospective case series [25] comprising 2,500 unrelated cases referred for LQTS clinical genetic testing.

For the five LQTS genes with case series data, Table 1 shows that the two approaches yield different estimates, as the burden of pathogenic variants in individuals referred for LQTS genetic testing in the clinical case series (30%) was lower than that typically reported in research studies (70% to 75%) [21,22]. It is likely that clinicians employ different thresholds for defining cases in a research setting or subspecialist clinical centre than are employed for day-to-day referrals to a clinical genetic diagnostics service. For better performance in the latter, more challenging, setting, we based our rare variant burden estimates on the case series. Given that these estimates were on average about half those predicted from the literature when both estimates were available, we reduced by a factor of two the prior odds derived from literature-based estimates for the other eight LQTS genes (*ANK2*, *KCNJ2*, *CACNA1C*, *CAV3*, *SCN4B*, *AKAP9*, *SNTA1* and *KCNJ5*).

Prior odds for HCM- and BrS-related genes were estimated using only information from the literature, in the absence of additional prospective case series for these syndromes, as summarised in Table 1. Combining literature-derived case burdens with population-derived estimates of control burdens led to unstable estimates of model parameters, due to the small numbers of variants. To avoid widely fluctuating priors (with very low confidence), we used an average estimated benign rare variant burden of 1% for these genes, yielding conservative priors.

### Predictors of pathogenicity

Having established prior odds for individual genes for each syndrome, we next consider sources of evidence that can be used to obtain the posterior probability of pathogenicity for an observed variant. First we describe the training data, comprising rare variants of known pathogenic/benign status, which will be used to estimate model parameters.

#### Training data

We generated a compendium of previously reported sequence variants in our genes of interest from HGMD professional version 2011.4, UniProt, dbSNP 135 and published case series [5,12,25]. From these we selected a subset of variants that could be robustly categorised as benign or pathogenic, assessed separately for each syndrome (LQTS, BrS and HCM). Variants were defined as pathogenic if they were reported as causing disease with supporting evidence of a functional effect *in vitro* and/or mechanistic information. Variants were defined as benign if identified in prospectively ascertained cohorts of healthy individuals [25], or if present in any dbSNP population with a frequency >0.01. Variants not satisfying either of these criteria, and hence assessed to have intermediate probability of pathogenicity, were removed. In total, 320 variants were retained in the LQTS training set (164 pathogenic and 156 benign), 73 variants in the BrS set (17 pathogenic and 56 benign), and 95 variants in HCM-related genes (67 pathogenic and 28 benign). These comprised 428 missense variants, 41 radical and 19 inframe indels. Variants are shown in Tables S1,S2,S3 (Additional file 1).

For HCM genes, we also used a second, independently ascertained training set. Pathogenic variants were taken from the training set previously applied to the PolyPhen-HCM classifier [12], supplemented with further pathogenic variants manually curated to the same robust criteria, and additional benign variants identified in a well-phenotyped control cohort [6]. These sources yielded 18 pathogenic and 39 benign substitution variants.

#### Protein domains

The pathogenicity of a coding variant depends on its location within the protein. Clustering of rare coding variants in hotspots has been described for a number of proteins [26]. This may simply represent high local mutability and/or lack of constraint, if variants cluster in both cases and controls. Alternatively, the region may be intolerant of variation, and an increase in the proportion of variants that are pathogenic may be informative for the interpretation of subsequent variants [5,16].

Therefore, we included domain-specific terms, with uninformative prior distributions, in the linear predictors for inframe and missense variants. Each domain effect is assumed to be normally distributed, with mean zero and variance an inverse-gamma random variable. For each gene, protein domain annotations were obtained from Uniprot [27] using the ‘Regions’ fields of the Sequence Annotation section. We explored informative prior distributions for domain terms; for example, estimates of the probability of pathogenicity of variants found in distinct domains of *KCNQ1*, *KCNH2* and *SCN5A* in [5] were considered. However, informative priors did not improve predictions, probably because these correlated with domain effects estimated *de novo* from our LQTS training data, which included many of the variants used by Kapa *et al.* to derive their estimates. No domain term is included for the prediction of variants in genes *KCNJ2*, *CAV3*, *SCN4B*, *AKAP9*, *SNTA1*, *KCNJ5*, *CACNA2D1*, *CACNB2*, *GPD1L*, *SCN1B*, *MYBPC3* and *TNNT2*, because domains of these genes are not sufficiently represented in our LQTS, BrS and HCM training data.

#### Allele frequencies in controls

Rare monogenic diseases cannot be attributed to variants that are common, though such variants may contribute to the risk of common complex diseases, or act as modifiers of rare disease. However, pathogenic variants may exist at a low frequency in populations of ostensibly healthy controls, as these may be in a pre-clinical disease phase, or phenotyped using methods with incomplete sensitivity. We explored the use of variant frequency as a quantitative predictor, but found that the best performance was obtained using a binary indicator of whether the variant has been recorded in a control database. As control sets improve over time, this decision may need to be revisited.

Control data sets were taken from the 1000 Genomes project and the Exome Sequencing Project [20,28]. The 1000 Genomes frequency was obtained from phase 1 of the project (2,184 haploid exomes) and comprised data from 14 distinct populations including European (five), African (three), East Asian (three) and Latin American (three) populations. The Exome Sequencing Project frequency was derived from approximately 13,000 haploid exomes from European-Americans and African-Americans in a 2:1 ratio. Published variants from a further control cohort of seemingly healthy controls and volunteers [5] comprising 1,300 individuals (47% Caucasian, 26% African American, 11% Hispanic, 10% Asian and 6% unknown/other), were used for the three major LQTS genes (*KCNQ1*, *KCNH2* and *SCN5A*). For our LQTS training data, 40% of the benign variants had a non-zero frequency in the controls, in contrast to 2% of pathogenic variants (typically only one occurrence).

#### Conservation

Where a protein residue is conserved across a wide range of species, this is widely interpreted as evidence that the residue is functionally important and hence intolerant of variation. We used protein sequence alignments of Ensembl-defined one-to-one orthologues for up to 70 species, and for each variant we recorded the species in which the corresponding residue was conserved. Due to the variable quality in genome sequencing and the automated nature of the orthologue alignments, a residue was deemed as conserved if it aligned with the same amino acid and non-conserved if aligned with a different amino acid; for residues aligning with gaps in the alignment or X (indicating poor sequence quality), the data for that species were disregarded. Residues were initially classified into five non-overlapping categories indicating whether they were conserved in all 70 species, all vertebrates, (eutherian) mammals, primates or were not conserved. During model fitting, this was simplified to three categories of conservation, encoded as two binary indicators (conserved in primates and conserved in all species) with not conserved as the reference state.

#### Non-synonymous single nucleotide polymorphism algorithms

Several algorithms aim to predict the pathogenicity of missense substitutions, largely based on physicochemical properties of the reference and alternate amino acids and their sequence contexts. Three of these have been incorporated as predictors in our model.

The Grantham score [29], first described in 1974, is a measure of the physicochemical similarity of pairs of amino acids. It is not dependent on sequence context. Polymorphism phenotyping v2 (PolyPhen) aims to predict the impact of single amino acid substitutions using sequence, phylogenetic and structural information [30]. It is available in two flavours, trained on different data sets. HumDiv aims to identify alleles that may alter protein function, while HumVar aims to discriminate variants with large effects (sufficient to cause Mendelian disease) from alleles that are at most only mildly deleterious. Our model uses the HumVar classifier. The sorting intolerant from tolerant (SIFT) algorithm also predicts whether an amino acid substitution affects protein function, based on the degree of conservation of amino acid residues in sequence alignments derived from closely related sequences [31]. Importantly, SIFT does not predict whether an alteration in protein function will be sufficient to cause disease.

**Figure 1:**
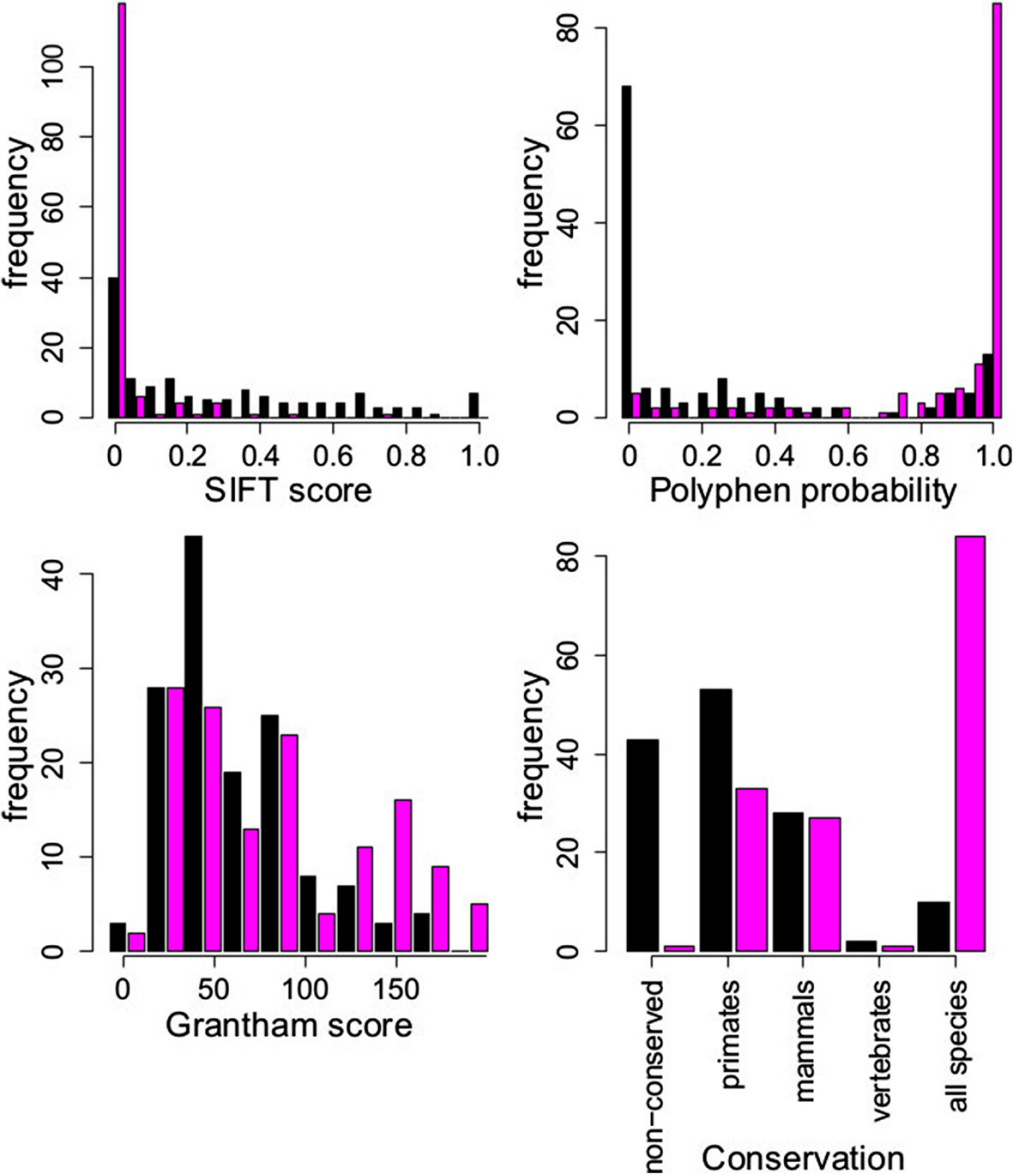
**Comparison of the distributions of predictor variables for benign and pathogenic variants.** Histograms depict the numbers of variants that are pathogenic (magenta bars) and benign (black bars) with predictor values within a range as indicated on the *x*-axes for the four predictor variables. Conservation categories are defined in the Methods section.

The distributions of Grantham, PolyPhen and SIFT scores obtained for pathogenic and benign variants in the LQTS training set are shown in Figure 1. The PolyPhen and SIFT scores both lie between 0 and 1, but with opposite polarities (higher PolyPhen scores and lower SIFT scores both indicate greater risk of pathogenicity). Grantham scores range up to 205 in the training set, with higher values indicating greater risk of pathogenicity. To facilitate interpretation of model coefficients in our model, Grantham scores were rescaled to lie between 0 and 1 by dividing by 205, and SIFT was replaced by 1−SIFT.

### Constructing the Bayesian prediction model

**Figure 2:**
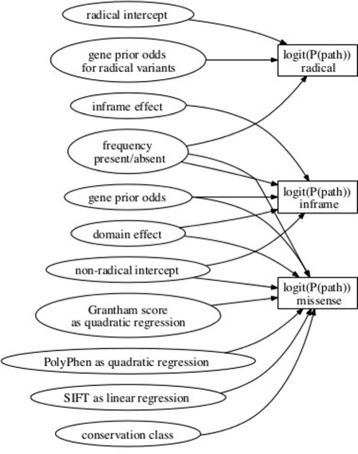
**Graphical representation of the three prediction models for a single syndrome.** The logistic regression models are represented as the three rectangles on the right, for a radical variant (top), an inframe indel (middle) and a missense substitution (bottom). Ellipses describe model predictors. Each model is additive on a logistic scale. Multiple arrows emerging from an ellipse indicate that the parameter is shared across the models indicated by the destinations of the arrows. This diagram represents the model for one syndrome.

Our Bayesian logistic regression model is represented diagrammatically in Figure 2, and in BUGS format in Additional file 2. For each variant, the logistic transform of the probability for it to be pathogenic is calculated as a sum over contributions from the predictor variables described above. The contributions from quantitative predictors are either linear or quadratic.

Our model proposes interrelated linear predictors for each of the three classes of variant, chosen because of background information indicating different probabilities of pathogenicity associated with each class. While the majority of cases of inherited cardiac conditions are caused by missense substitutions, there is also an appreciable burden of rare missense variants in healthy controls [5,25]. By contrast, radical variants are almost absent from the general population, so that for many inherited cardiac conditions such a variant in a patient is conventionally considered pathogenic [5]. Inframe indels have previously been classified as radical, because they are also rare in healthy controls [5], but we have classified these separately because of the evidence for different properties of this class [25]. The likely effects of different variant classes also depends on the molecular mechanism of disease. A missense variant may lead to either loss or gain of function, whereas radical variants usually produce loss of function. The probability of pathogenicity of radical variants should depend on whether disease is mediated by loss or gain of function of the gene of interest. Non-coding variants and near splice site substitutions that do not disrupt the consensus donor/acceptor sites are not considered in the model.

The model for radical variants is the simplest, including only two predictors (variant frequency indicator and gene effect). The intercept represents the average pathogenicity of radical variants (Figure 2) and it, like other intercept parameters introduced below, is assigned a *N*(0,10) prior distribution. Domain terms are not included because the fraction of radical variants that are pathogenic is similar across domains [5], and loss of function is expected to be largely independent of the precise location of the truncating variant. Conversely, gene effects are important for the pathogenicity of radical variants due to the differences between genes in the effects of gain-of-function and loss-of-function variants, among other factors.

The model for inframe indels is similar to that for radical variants, but also includes domain-specific terms. Domain terms have normal prior distribution centred at 0 (its variance is estimated from the data and has an inverse-gamma prior distribution). We use the same gene effects as for missense variants. For interpretability of the parameters, we also include the intercept that measures average pathogenicity over all non-radical variants, and we add to this the inframe effect term, which measures any excess pathogenic risk of inframe variants over missense substitutions.

The richest model is for the missense substitutions, which includes all the predictors mentioned above plus Grantham, SIFT, PolyPhen and the two conservation indicators, for primates and all species. For the three quantitative predictor variables, we considered various models in a series of simulation experiments and found the best performance with SIFT modelled as a linear covariate, but Grantham and PolyPhen scores both included as quadratic functions. The linear part of each model is assigned a *N*(0,10) prior distribution.

Our model uses several hyperparameters. One hyperparameter determines the standard deviation of a normal prior. Two hyperparameters specify a gamma distribution that models domain effects. We have tried experimentally varying the values of these parameters. When we replaced standard deviations of normal distributions by larger values, for example 100, we found that the fitted effect sizes of predictors became large, leading to overfitting. In contrast, smaller standard deviations shrank the effect sizes too much, thus preventing confident predictions for many variants. The parameters of the gamma distribution had a less noticeable effect on the results; in the end we chose parameters that are consistent with our expectations of the magnitude of domain effects and that prohibit overfitting, which is a potential concern with nearly 60 domains of LQTS genes.

#### Model fitting and assessment

All model parameters are fitted simultaneously over the three variant classes to allow simultaneous estimation from the training data set of the shared parameters indicated by multiple arrows in Figure 2. Estimation for LQTS and BrS models was also done jointly for combined estimation of the shared parameters (variance of domain effects, scale of gene priors, intercepts for all variant classes and parameters modelling the effect of Grantham, SIFT, PolyPhen and conservation). The HCM model was fitted separately and also in combination with the LQTS model (the shared parameters were a subset of those shared for LQTS and BrS: variance of domain effects and parameters modelling the effect of Grantham, SIFT, PolyPhen and conservation).

The fitting was done by a Markov chain Monte Carlo (MCMC) procedure within the JAGS software [32]. Ten chains, each with 40,000 iterations, were simulated to estimate the effect sizes. The first 20% of outputs were discarded as burn-in. The convergence of simulations was assessed by a potential scale reduction estimator [33] applied to each parameter separately:

$$R = \sqrt{\text{Var}(\phi | y) / W} $$

where

$$\text{Var}(\phi | y) = W \times (n - 1) / n + B \times 1 / n, $$

while *B* is a variance in sampled parameter values between ten chains, *W* is a variance in sampled parameter values within a single chain and *n*=32, 000 is the number of non-discarded simulations. We required *R*<1.1 to terminate, which was always satisfied after 40,000 iterations.

The post burn-in simulations were used to report parameter medians, which were subsequently used as fitted values in a prediction model for novel variants. Posterior probability distributions for the model parameters can be estimated from the MCMC output, which is useful for model diagnosis. Goodness of fit for all models was assessed via cross-validation. We randomly split the data set 100 times into training and test sets such that each test set had 1/10 of pathogenic and 1/10 of benign variants. We fitted the model (Figure 2) to each training set and then calculated the pathogenic risk for the test variants.

Although the model outputs a posterior probability of pathogenicity that is continuous and innately interpretable, we also assess model performance by applying a threshold to probabilities of pathogenicity and calculating standard metrics including sensitivity, specificity and positive predictive value (PPV). To ensure that the PPV appropriately reflects the prevalence of variants in the intended test population (rather than the proportion of benign and pathogenic variants in the cross-validation test set), we calculate PPV using the equation:

(5) $$ {\fontsize{9}{12}\begin{aligned} \text{PPV} = \frac{\text{Prior odds} \times \text{True positive rate}}{\text{(Prior odds} \times \text{True positive\ rate)} + \text{False positive rate}} \end{aligned}}  $$

where the prior odds are derived for each syndrome as described above and summarised in Table 1. Recall that the true positive rate is synonymous with sensitivity, and:

$$\text{False positive rate} = 1 - \text{Specificity} $$

A model description in BUGS format, a table of fitted model coefficients, and scripts necessary to reproduce these analyses are provided in Additional files 2, 3, 4.

## Results

### Models for long QT syndrome

The results for 100 cross-validation data sets show that most of the pathogenic variants were assigned high probabilities of pathogenicity: 73% of them are assigned *P*(pathogenic)>0.9, while 2.2% are assigned *P*(pathogenic)<0.1. Conversely 71% of benign variants are assigned *P*(pathogenic)<0.1, while only 0.6% are assigned *P*(pathogenic)>0.9.

Note that we are using *P*(pathogenic), the probability that a variant is pathogenic, as shorthand for *P*(pathogenic|disease status+variant data), the probability that a variant is pathogenic if it has certain characteristics and given that it is found in an individual with an appropriate disease phenotype.

**Figure 3:**
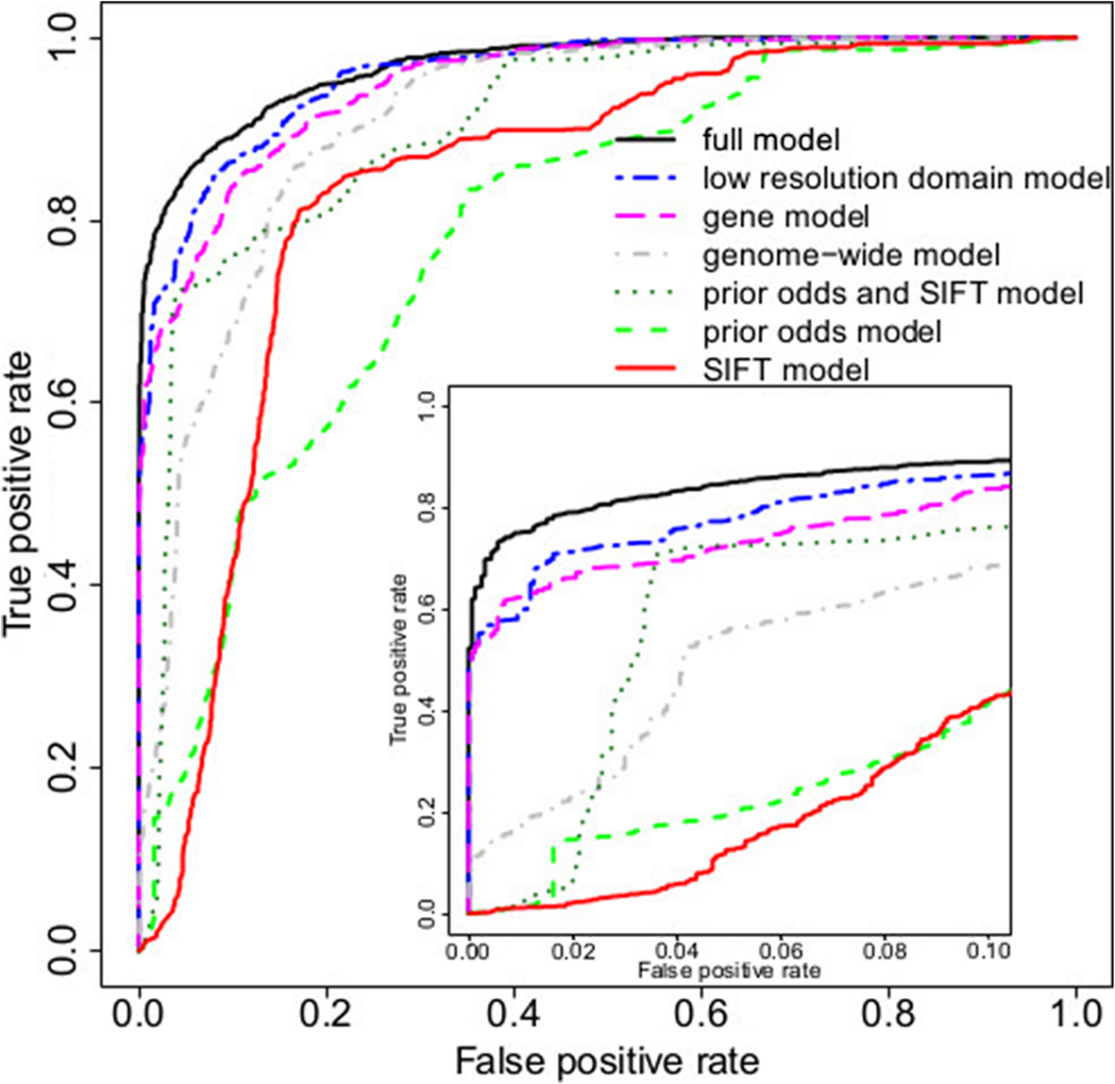
**Comparison of pathogenicity prediction models for LQTS.** Receiver operating characteristic curves are shown for four nested LQTS models, as well as for SIFT with and without the addition of prior odds. The inner plot shows the false positive rate from 0 to 0.1, while the axis of the outer plot spans the false positive rate from 0 to 1. See text for explanation of the models. LQTS, long QT syndrome.

Figure 3 depicts the receiver operating characteristic (ROC) curves for this model, using predictions for 3,200 test variants from the 100 data splits, and Additional file 1: Figure S2 shows alternative representations of model performance. Ranking variants according to their probability of pathogenicity, the top 84 places are taken by pathogenic variants (53% of pathogenic variants). The PPV associated with a particular threshold for *P*(pathogenic) depends on the prior odds. From Table 1, prior odds were 45 for *KCNQ1*, 25 for *KCNH2* and 4 for *SCN5A*, giving overall prior odds of 25 for these three genes. If we choose a threshold of *P*(pathogenic)>0.9, then the combined PPV for *KCNQ1*, *KCNH2* and *SCN5A* is 0.999. At this threshold, 79% of pathogenic variants in *KCNQ1*, 82% of *KCNH2* and 39% of *SCN5A* were identified, with a combined sensitivity of 76% across the three genes. For the other genes that much more rarely cause LQTS, sensitivity is lower (25%), but the PPV remains high (1). As the training data are relatively sparse for these genes, predictions are appropriately cautious. Assessing across all LQTS genes, the combined sensitivity is 73%, with PPV >0.999. Performance metrics at other thresholds are shown in Additional file 1: Figure S2 and Table S5.

The sensitivity of predictions can be different for the three variant classes: 96% of pathogenic radical variants are assigned *P*(pathogenic)>0.9 while none of the pathogenic inframe indels is assigned high probability of pathogenicity. More cautious predictions for inframe indels might be caused by the fact that most of the inframe indels in the LQTS data set are benign. 71% of pathogenic missense substitutions are assigned *P*(pathogenic)>0.9. Interestingly enough, the specificity is very high for missense substitutions: only 0.07% of benign variants are assigned *P*(pathogenic)>0.9. For three major LQTS genes (*KCNQ1*, *KCNH2* and *SCN5A*), sensitivities across the three variant classes are as follows: 75% for missense variants, 96% for radical variants and 0% for inframe indels. Similar sensitivities are obtained across all 13 genes. PPVs for the three major genes are 0.999 for missense variants, 1 for radical variants and 0 for inframe indels.

#### Optimising model predictors for long QT syndrome

We explored the choice of model predictors by comparing nested submodels of the model shown in Figure 2, to determine whether the model could be simplified without loss of predictive accuracy. The models considered are:

- A full model as shown in Figure 2.
- A low-resolution domain model in which domain annotations are simplified by merging domains to yield a coarser description of the domain structure. This involved merging the six transmembrane helical domains and intervening extra- and intracellular segments into transmembrane regions and looking at the entire N- and C-termini regardless of specific functional domains (PAS/PAC/cNBD in *KCHN2* and SAD in *KCNQ1*).
- A gene model, without domain effects.
- A genome-wide model with neither gene nor domain effects.

For comparison against existing methods we also quantified the performance of SIFT on the same data set, with or without gene-specific priors:

- A prior odds and SIFT model contains only prior odds of genes, SIFT effect and a separate intercept for each variant class.
- A prior odds model distinguishes variant classes and uses prior odds of genes.
- A SIFT model where SIFT is the only predictor apart from a variant class.

The low-resolution domain model is consistently less accurate than the full model (Figure 3). It appears that including individual domains in the model is useful, despite the fact that the data are sparse for some domains. The gene model reaches a true positive rate of about 75% for a false positive rate of 5%. It is inferior to the full model at the small false positive rates that are of interest in practice. The genome-wide model is similar to existing genome-wide pathogenic risk predictors. The results obtained from this model are considerably worse than for the gene model, indicating that the incorporation of prior odds of pathogenicity for individual genes has a major impact on predictive accuracy. The prior odds and SIFT model occasionally outperforms the genome-wide model, but is worse at small false positive rates. Both the prior odds model and the SIFT model achieved 40% sensitivity at a 10% false positive rate. Neither of these predictors on their own could make a model that would have sufficient sensitivity and specificity; however, combining the prior odds of individual genes and SIFT score noticeably improved the predictive performance relative to either alone.

### Models for Brugada syndrome

**Figure 4:**
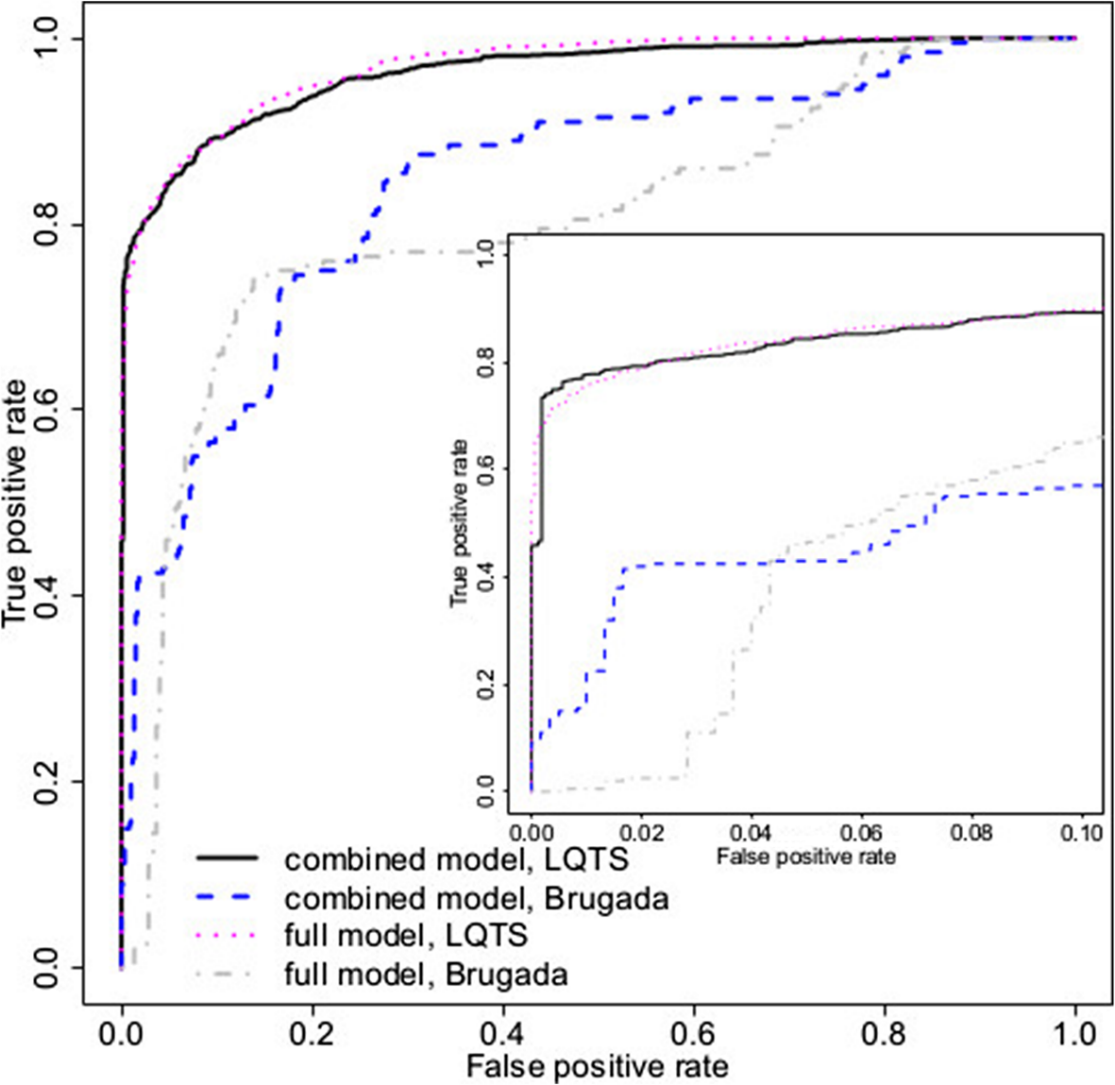
**Pathogenicity prediction models for Brugada syndrome.** The receiver operating characteristic curve for the full model for BrS is shown alongside that for LQTS (as in Figure 3) for comparison. Sensitivity could be improved at low false positive rates by building a combined model, in which some parameters were fit jointly for the LQTS and BrS models (see text for details) to compensate for the smaller BrS training set. Joint fitting does not impede performance of the LQTS model. BrS, Brugada syndrome; LQTS, long QT syndrome.

The results in Figure 4 show that the sensitivity attained by the initial full model is close to 65% at a false positive rate of 10%, increasing to 75% at a false positive rate of 20%. Simpler models, excluding gene or domain effects, were inferior as for LQTS, and are not shown. In the magnified panel, the curve is nearly horizontal near the origin (false positive rate 0 - 0.03) due to a few benign variants having *P*(pathogenic)≈1. A closer inspection revealed that two missense variants had a high probability of pathogenicity due to a specific combination of predictors: large PolyPhen probability, SIFT score close to 0, frequency absent and a moderate to large Grantham score. Also one benign radical variant in *SCN5A* had *P*(pathogenic)≈1: there was no frequency data for it.

We compared the predictive performance of a full model for BrS and a gene model for BrS that has no domain effects. Both sensitivity and specificity were lower for the gene model, and the differences between both models were similar to the differences between equivalent models for LQTS. In addition, we ran simulations for a genome-wide model in the context of BrS: the predictions yielded by a genome-wide model were less accurate than those of the gene model and again the differences between models were similar to those observed for LQTS.

We tried to enhance the predictions for BrS by combining the training data set with the LQTS data set. We postulated a new multivariate model with two outcomes (pathogenic/benign for LQTS and pathogenic/benign for BrS), which was then trained on the combined data. Gene and domain effects were estimated separately for LQTS and BrS, but the effects of SIFT, PolyPhen, Grantham, conservation, frequency and inframe variants were estimated jointly across the outcomes. Intercepts and scale parameters for prior odds of genes were also shared across the syndromes, as the small number of radical and inframe variants in the BrS test set did not permit robust estimation of these parameters. Each of the 100 cross-validation test sets included 1/10 of the pathogenic variants and 1/10 of the benign variants for each of LQTS and BrS. The ROC values were computed for each syndrome separately, using only the test-set variants associated with that syndrome.

The combined model improved the BrS predictions for false positive rates smaller than 5% (Figure 4 and Additional file 1: Figure S2). The number of benign variants with high probabilities of pathogenicity has fallen, despite there being few training variants in some BrS genes. The separate (full) BrS model performs better at false positive rates between 5% and 20%, which is of less clinical usefulness. Under both models, the prediction accuracy for BrS remains inferior to that of LQTS, because of limited data. The performance for the LQTS variants remained the same.

For *SCN5A*, the prior odds were 30 for BrS referrals, and the PPV for the *SCN5A* gene at a threshold of *P*(pathogenic)=0.9 was 0.963 for the full model and 0.998 for the combined multivariate model. Only 2.6% of pathogenic variants could be detected with threshold 0.9 when the full BrS model was used, while the sensitivity increased to 23% for the same threshold in the multivariate model (sensitivity comparison for other thresholds is summarised in Additional file 1: Figure S2 and Table S5). However, the proportion of confident predictions is considerably lower than for LQTS due to the smaller training set.

### Models for hypertrophic cardiomyopathy

Finally we built a prediction model for HCM based on rare variants in *MYH7*, *TNNT2*, *TNNI3* and *MYBPC3*. We had two training sets available for this syndrome (see above). After checking that the predictive models built on each data set separately performed similarly, we merged the training sets, resulting in 85 pathogenic and 67 benign variants, after removing 14 duplicates. These were randomly allocated into 100 training and test sets as for LQTS.

**Figure 5:**
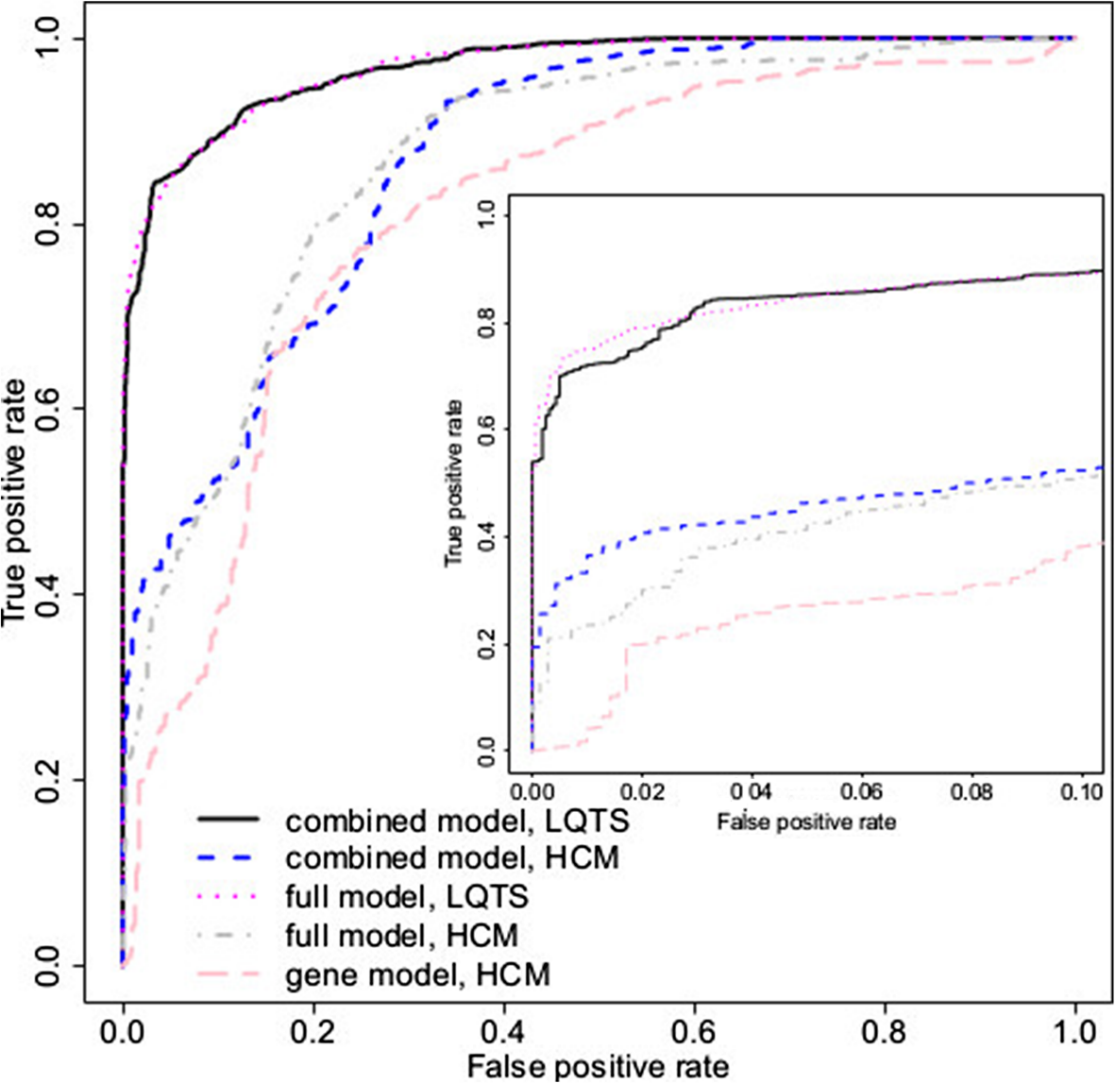
**Comparison of pathogenicity prediction models for hypertrophic cardiomyopathy.** Receiver operating characteristic curves for the full HCM model (full model, HCM) and a simpler gene model without domain-specific prediction (gene model, HCM) are shown alongside the LQTS classifier for comparison. As for BrS, the full HCM model was re-estimated in combination with the LQTS model with modest benefits at low false positive rates. BrS, Brugada syndrome; HCM, hypertrophic cardiomyopathy; LQTS, long QT syndrome.

We compared the full model with a gene model that omitted domain effects. The full model is shown in Figure 5, alongside the full LQTS model for comparison. The full model reached sensitivities of 40% and 50% with false positive rates of 5% and 10%. The gene model is inferior to the full model for any false positive rate considered.

As before, we investigated merging the HCM and LQTS models to improve predictive performance. Merging was as described for the BrS model, except that intercepts and scale parameters of prior odds of genes were estimated separately for HCM and LQTS, as the HCM data set was larger than that for BrS. The ROC curves were drawn for each syndrome separately (see Figure 5 and Additional file 1: Figure S2).

As for BrS, combining data across syndromes improves predictions at low false positive rates, but it remains that high sensitivity for HCM can only be achieved with large false positive rates. The relatively poor performance for the HCM model, with or without merging, may be attributable to a less informative domain architecture for HCM genes, and relatively more missense substitutions and fewer radical variants in the HCM training data.

We calculated sensitivities and PPVs for the predictions for variants in HCM genes when the combined multivariate model is used. The PPV for *MYH7* at threshold 0.9 is 0.994, but only 26% of pathogenic *MYH7* variants are assigned probabilities >0.9. For *MYBPC3*, no pathogenic variant had a probability of pathogenicity >0.9 (see Additional file 1: Table S5). The multivariate model was surprisingly sensitive when detecting pathogenic variants in *TNNT2*: 83% of these variants had probability >0.9, while the PPV was 0.985 at this threshold. All PPVs for *TNNI3* were 1, although sensitivity was rather low: 34% of pathogenic variants were identified at the 0.9 threshold. The variability of sensitivity across HCM genes was unexpected and could probably be eliminated by more detailed domain annotations.

### Estimated effect sizes of predictors in the long QT syndrome model

**Figure 6:**
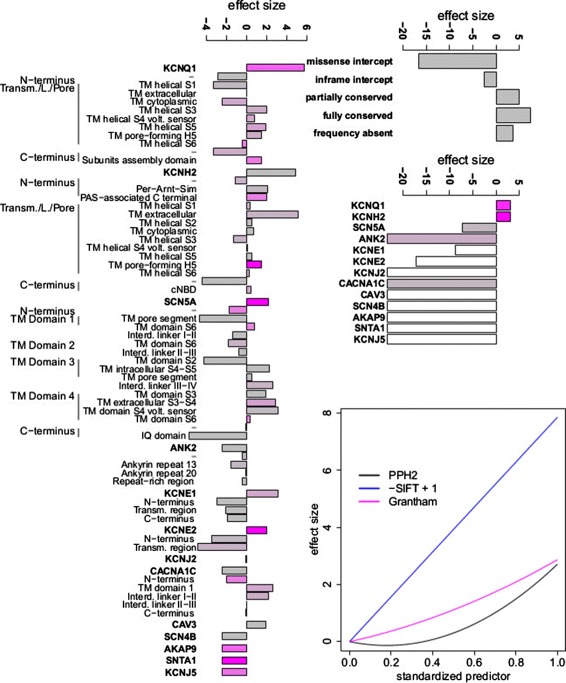
**Magnitude of effect sizes estimated from the full LQTS model.** A positive effect size indicates that the evidence supports pathogenicity, whereas a negative effect size indicates evidence against pathogenicity. On the left, the effect size for each gene and domain term is shown, with gene effects corresponding to the logarithm of the prior odds for non-radical variants in Table 1 multiplied by a scale parameter. Domains are ordered sequentially according to genome position. The colour of the bars reflects the number of variants in the LQTS training set for each gene or domain: magenta bars are derived from many training variants (indicating high confidence), and grey bars from few variants. The top right panel shows the effect size for other binary variables, i.e. variant class (inframe/missense), allele frequency and conservation classes. The middle right panel includes gene terms for radical variants, where a gene term corresponds to the logarithm of the prior odds for radical variants in Table 1 multiplied by a scale parameter and added to the effect of variant class (radical). Magenta bars imply many training variants, while grey bars indicate few radical variants and white bars denote genes without any radical variants in the LQTS training data. The bottom right panel shows the effect size for continuous predictors (nsSNP algorithms) as linear or quadratic functions of the predictor value. Interd., Interdomain; IQ, IQ calmodulin binding motif, named after the first two amino acids of the motif, isoleucine (I) and glutamine (Q); L., Linker; LQTS, long QT syndrome; PAS, Per-Arnt-Sim domain, named after homology to the Drosophila period protein (PER), the aryl hydrocarbon receptor nuclear translocator protein (ARNT) and the Drosophila single-minded protein (SIM); PPh2, PolyPhen-2; TM, Transmembrane; Transm., Transmembrane; volt., voltage.

Focussing on the LQTS model because of the much larger training set available, we investigated which predictors are the most informative, and assessed the biological plausibility of estimated effect sizes (Figure 6). For all predictors for which there was a clear prior expectation, the directions of effects are as expected; for example, the probability that a variant is pathogenic increases with its level of conservation.

SIFT score has a large effect on predictions (Figure 6), while PolyPhen and Grantham each have a moderate impact. The quadratic influence upon the pathogenic risk is more pronounced for PolyPhen than for Grantham when modelled jointly with other predictors, whereas assessed individually they showed an equally strong indication of non-linearity.

The intercept for missense variants (Figure 6) is highly negative, reflecting their greatly reduced probability to be pathogenic relative to inframe indels and radical variants, and so substantial other evidence is required for a missense variant to have high probability of pathogenicity. The inframe intercept is much higher but somewhat less than that for radical variants, in concordance with previous reports [5].

The gene effects for radical variants imply that the predictions for this type of variant are confident in both directions: variants in two major LQTS genes are classified as pathogenic, while others are assumed to be benign in the absence of other evidence. This is consistent with molecular mechanisms underlying LQTS caused by these genes, for example LQTS is caused by gain of function of *SCN5A* and *CACNA1C*, while radical variants are likely to cause loss of function. The small number of radical variants for all LQTS genes apart from *KCNQ1* and *KCNH2* leads to a considerable weight being given to the prior odds of genes for radical variants by the model fitting.

The domain effects in Figure 6 are grouped by genes and ordered according to the sequence of domain coding segments on the genome. There is evidence of clusters of domains with similar magnitude and direction of effect, in keeping with a previous report [5]. For example, consistent domain effects are observed for *KCNQ1* (from the Transmembrane/Linker/Pore region and TM helical S3 domain through to the end of the Transmembrane/Linker/Pore segment), for *KCNH2* (from the N-terminus (Per-Amt-Sim) till the Transmembrane/Linker/Pore (TM cytoplasmic domain)) and for *SCN5A* (from TM domain 3 (TM intracellular S4-S5) until the end of TM domain 4). No prior information about domain order was used by the model, and this observation suggests that there is scope for better predictions that exploit domain order. However, simplifying the model by merging domains in the low-resolution LQTS model reduced prediction accuracy, perhaps because boundaries of the observed domain clusters do not always coincide with the UniProt features that we used as the basis of our mergers [5]. For example, in *KCNQ1* a boundary emerges within the Transmembrane/Linker/Pore region, between a TM cytoplasmic domain and the helical S3 domain, rather than at the limit of the Transmembrane/Linker/Pore region. Our unexpected observed boundaries on further investigation might lead to improved understanding and better predictions.

Although domain effects were estimated from information about variants in the training set alone, many of these effects are consistent with the literature. For example, it has been reported that variants in Transmembrane/Linker/Pore domains of *KCNH2* are more likely to be pathogenic than those in either the C-terminus or N-terminus [5]. This is confirmed in Figure 6, which also yields more detailed information about the pathogenic risk of individual Transmembrane/Linker/Pore domains.

### Model implementation and distribution

We have developed an online implementation of the full prediction model for LQTS: APPRAISE: Assessing Pathogenicity PRobAbility by Integrating Statistical Evidence [34]. Scripts and materials required to replicate these analyses are provided in the Additional files, and are available on GitHub [35].

## Discussion

We present a network of nine interrelated Bayesian logistic regression models that discriminate between pathogenic and benign variants in genes associated with inherited cardiac conditions. The nine models encompass three variant classes (radical, inframe indel and missense substitution) for each of three syndromes (LQTS, BrS and HCM). A feature of our models is the gene- and domain-specific terms, which are estimated from training data specific to each syndrome. Another feature is the sharing of some predictors across syndromes, to benefit from improved parameter estimation by combining training data over syndromes. Our approach should be widely applicable to other monogenic inherited conditions, provided that suitable training data are available, but the extent to which information can be shared across syndromes requires careful investigation.

The output of this Bayesian approach is a posterior probability of pathogenicity, allowing users to choose their preferred threshold for declaring a variant to be pathogenic. In the numerical examples here, we have taken a probability of 0.9 as a threshold value to calculate performance metrics for comparison. Other categories can also be defined by appropriately partitioning the probability values, for example ‘possibly pathogenic’ or ‘likely benign’ [9,30,31].

Our Bayesian framework is transparent in its assumptions and can be readily extended to incorporate new lines of evidence to assess the pathogenicity of a novel genetic variant. In particular, familial segregation of a phenotype with a variant provides evidence of pathogenicity that is independent of the predictors in our models. The LOD (logarithm of odds) score used to quantify linkage between a variant and a trait is a likelihood ratio, and the posterior probability can easily be recalculated in the light of this further evidence, e.g.

$${\begin{aligned} \text{Posterior odds} =&\; \text{Prior odds} \times \text{LR(predictor model)} \\ &\times \text{LR(segregation data)} \end{aligned}} $$

This means that modest evidence for pathogenicity may be combined with modest segregation data from a small family to yield a confident prediction.

In practice, the probabilities reported by our models are conservative because of the conservative prior probabilities that we have employed. For example, we found that using the cut-off *P*(pathogenic)>0.9 yields a PPV of 0.999, with sensitivity 0.76 combined over the three most important LQTS genes (sensitivity is 0.73 for all 13 LQTS-related genes). In other words, a probability of pathogenicity >0.9 represents a highly confident prediction in favour of pathogenicity, but the price of this high confidence is that nearly one-third of pathogenic variants will be missed. Users of the model can choose other cut-offs to obtain a different balance between sensitivity and specificity.

Under what circumstances are confident, and therefore clinically actionable, predictions obtained? The gene effect sizes for radical variants imply that the predictions for novel radical variants in these genes will almost always be confident, in keeping with our prior knowledge, and in accordance with current clinical practice. Inframe indels have previously been treated as radical [5], though there are a number of polymorphisms in this class. The use of frequency information in the model allows for confident benign predictions for this type of polymorphism. Novel missense substitutions overall yield less confident predictions, as expected. Nonetheless, the use of domain information in particular allows for justifiably confident predictions for variants in some protein domains. Strong and concordant predictions from SIFT and PolyPhen can also generate a relatively high probability of pathogenicity for missense substitutions in some genes.

One aspect of our decision-making that is made explicit in this framework is the prior probability of pathogenicity. Here, we have estimated this on a gene-by-gene basis for each syndrome. Importantly, when we compared a series of nested models of increasing complexity, the single largest improvement came in moving from a genome-wide model to a gene-specific model, suggesting that this approach, tailored for each gene and syndrome, is more powerful than genome-wide approaches, as has been suggested previously [12,13]. This suggestion can be confirmed as more data become available through centralised databases and registries.

Our prior probabilities are based on prospective real-world estimates of the yield of genetic testing, rather than curated case series that selectively include only robustly phenotyped cases. Nonetheless, if clinicians were to employ genetic testing without appropriate case selection, then the priors used here would no longer be applicable. However, the framework does provide an opportunity for further refinement, in which the prior probability could be modulated by the patient’s phenotype. For example, the prior probability might be augmented by extreme values of the QT interval in LQTS, by ventricular wall thickness in HCM or by a strong family history.

The priors can also be adjusted to allow for different testing strategies. The current priors assume that all genes for a given syndrome are examined simultaneously, for example, using a broad next-generation sequencing panel. Alternatively, one might choose to test only the three principal LQTS genes in a first wave of testing, and test the remaining genes only in those who are genotype negative, as is common practice in laboratories using conventional sequencing. Here the gene probabilities for the second round of testing depend on the results of the first: the probability that an observed variant in a minor LQTS gene is pathogenic is substantially higher if this testing is reserved for those with a clear phenotype, but no causative variant in the three principal LQTS genes.

Having concluded that gene-specific models are preferred to genome-wide models, it is notable that the models could nonetheless be improved by merging some parameters between models, particularly where training data were sparse. For example, the use of training data from several diseases to estimate the likelihood ratio distributions for SIFT, PolyPhen, Grantham and Conservation scores, while retaining disease-specific gene priors and domain models, improved predictions from the BrS and HCM models, at least at the extremes of the distribution of outcomes.

We have arrived at the proposed Bayesian model via model selection. We have compared the predictive performance of the models making use of various subsets of predictors. In principle, the model selection process could be replaced by model averaging or ensemble learning. However, the number of available predictors was not so large in our case to benefit from the variety of models included in an ensemble (frequent repetitions of a few models would be inevitable). We observed in simulations that it was relatively straightforward to decide whether a predictor should be included in the model or not: it either had no influence on the sensitivity and specificity or there was a consistent improvement in sensitivity or specificity. However, we might need to consider probabilistic approaches to model selection when more predictors of rare variant pathogenicity become available.

Training the full model for a LQTS data set required approximately one and a half hours on a desktop computer running the Unix CentOS operating system (four cores of 2.83 GHz each and 3.6 GB RAM). The time of training a new model looks linear for the number of variants in the data set. Extra care has to be taken for memory, because as the data set increases the number of estimated parameters whose values are stored throughout the simulation can also increase; for example, there can be more genes and domains to take into account. It seems that our modelling approach could be applied to data sets with a few thousand rare variants, if more powerful computational means are employed and additional optimisations for memory use are implemented.

This approach is limited in that variants are assessed individually for a likely causative role in monogenic disease. There is no consideration of oligogenic inheritance or the role of modifier variants. The severity of disease associated with any given variant is also not assessed. Rare variant burdens in cases and controls were not obtained using a uniform sequencing strategy, which could influence estimated prior odds. In our HCM model, we used the generic PolyPhen classifier, rather than the disease-specific PolyPhen HCM [12], because the PolyPhen HCM predictor was optimised using a small training data set that is incorporated into our HCM training data.

## Conclusions

Disease-specific Bayesian models provide a robust, accurate and transparent framework to integrate multiple sources of evidence available for the interpretation of novel variants identified during gene sequencing. The models presented here are immediately applicable to clinical diagnostics for inherited cardiac conditions, and provide important lessons for the development of predictors for other clinically important diseases.

## Additional files

Additional file 1
**Contains two figures and five supplemental tables.** **Figure S1**. Full prediction model for a single syndrome; it is a more detailed version of Figure 2 and specifies prior distributions for model parameters. **Figure S2**. Alternative measures of model performance. **Tables S1, S2, S3**. Variants included in the model training sets for the three syndromes studied. **Table S4**. Literature estimates of prior probabilities of pathogenicity for rare variants found in the genes studied. **Table S5**. Sensitivity and specificity of model predictions across a range of threshold values.

Additional file 2
**Model description in BUGS format (** http://www.openbugs.info).

Additional file 3
**Fitted model coefficients.**

Additional file 4
**Scripts and input files necessary to reproduce these analyses, also available at** http://github.com/jamesware/APPRAISE **.**

## Abbreviations

BrS: Brugada syndrome
HCM: hypertrophic cardiomyopathy
LOD: logarithm of odds
LQTS: long QT syndrome
LR: likelihood ratio
MCMC: Markov chain Monte Carlo
PPV: positive predictive value
ROC: receiver operating characteristic
SIFT: sorting intolerant from tolerant algorithm
