# Supplementary material for: Bayesian models for syndrome- and gene-specific probabilities of novel variant pathogenicity
Source: Genome Med. 2015 Jan 28;7(1):5. doi: 10.1186/s13073-014-0120-4 (PMC4308924; doi:10.1186/s13073-014-0120-4)
Supplement: Additional file 1 — Contains two figures and five supplemental tables. Figure S1. Full prediction model for a single syndrome; it is a more detailed version of Figure 2 and specifies prior distributions for model parameters. Figure S2. Alternative measures of model performance. Tables S1, S2, S3. Variants included in the model training sets for the three syndromes studied. Table S4. Literature estimates of prior probabilities of pathogenicity for rare variants found in the genes studied. Table S5. Sensitivity and specificity of model predictions across a range of threshold values. [file 13073_2014_120_MOESM1_ESM.pdf]

Supplemental Material

Supplemental figures

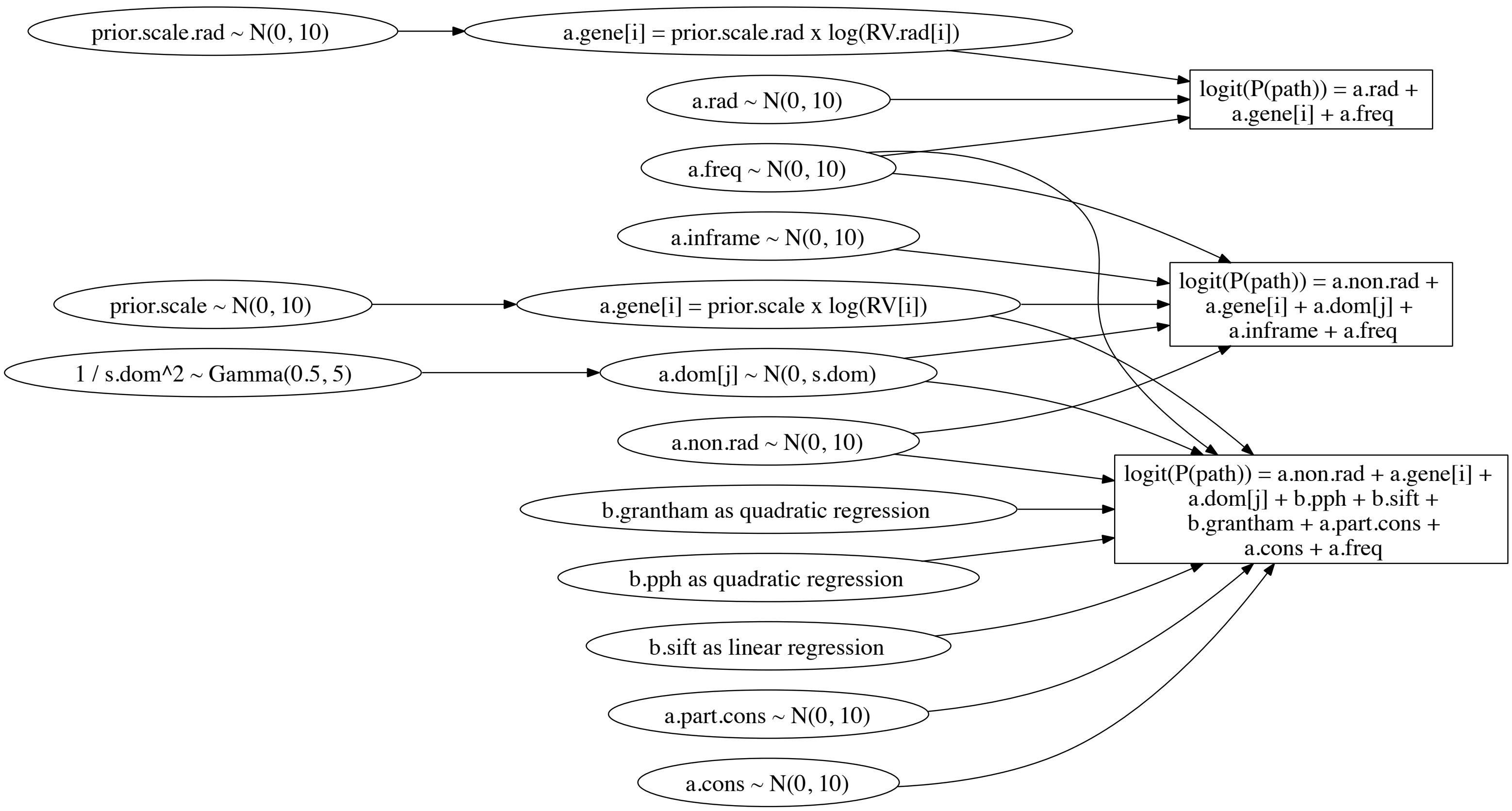

Figure S1: Detailed description of the three prediction models for a single syndrome. The logistic regression models are represented in the three rectangles on the right, for a radical variant (top), an inframe indel (middle), and a missense substitution (bottom) in domain j of gene i. Variables are not shown, only the corresponding regression coefficients: "a." indicates the coefficient of a binary variable and "b." that for a continuous variable. Ellipses describe prior distributions for these coefficients, and "s." indicates a standard deviation. Multiple arrows emerging from an ellipse indicate that the parameter is shared across the models indicated by the destinations of the arrows. This diagram represents the model for one syndrome. Explanation of functions and parameters follows. $N(\mu, \sigma)$: normal distribution with mean μ and standard deviation σ; Gamma(k, θ): gamma distribution with shape k and scale θ; a.non.rad: risk ratio for non-radical variants (also called non-radical intercept); a.rad: risk ratio for radical variants (radical intercept); a.inframe: risk ratio for inframe indels; a.gene[i]: risk ratio for gene i; RV[i]: rare variant burden ratio for non-radical variants in gene i (see Table 1); RV.rad[i]: rare variant burden ratio for radical variants in gene i; prior.scale: rescaling parameter of burden ratio; necessary when ratios are estimated from a cohort with either unknown or unusual prevalence of the disease; prior.scale.rad: rescaling parameter for the ratio of radical variants; a.dom[j]: risk ration for domain j; s.dom: standard deviation of domain effects; b.pph: PolyPhen regression term (see text); b.sift: SIFT regression term; b.grantham: Grantham regression term; a.part.cons: indicator of conservation in primates; a.cons: indicates conservation over all species.

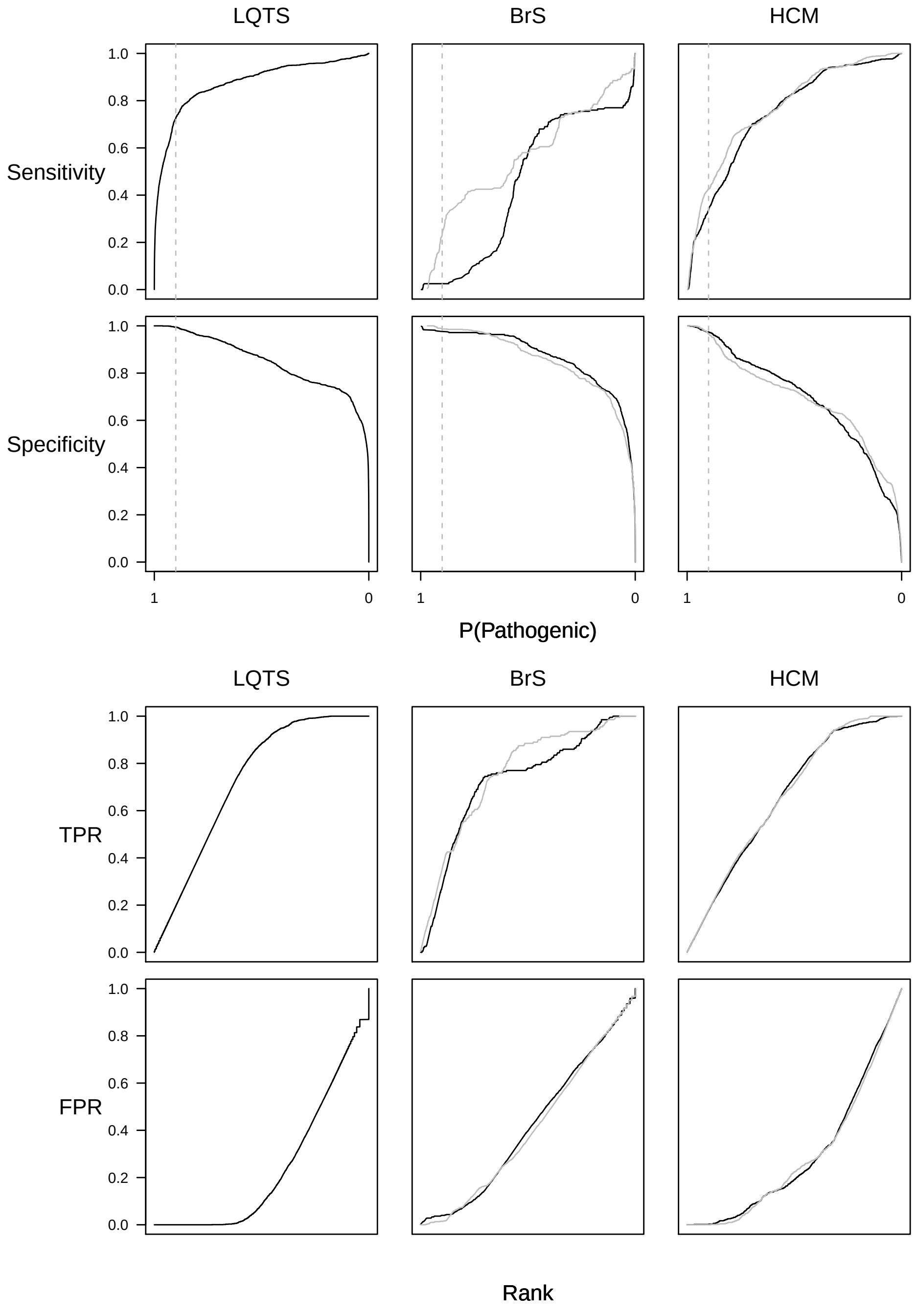

A

B

Figure S2: Assessing model performance. A. For each model, sensitivity and specificity are shown against a variable threshold for the probability of pathogenicity. A dotted line is shown at P(pathogenic)=0.9, used as the cut-off for performance metrics reported in the main text. As the threshold is decreased from 1, sensitivity increases and specificity decreases as expected. Black lines show the models trained using syndrome-specific training data (full model in Figs. 3-5), and grey lines represent models for BrS and HCM trained by jointly fitting some parameters across syndromes (combined model in figures 4-5). This joint fitting improves model performance due to pathogenic variants achieving high P(pathogenic) scores. B. For each model, cumulative true positive rate (TPR) and false positive rate (FPR) are plotted against the rank order of predictions. TPR = sensitivity = (number of pathogenic variants ranked $> x$) / total number of pathogenic variants. FPR = 1 - specificity = (number of benign variants ranked $> x$) / total number of benign variants. 52% of known LQTS-causing variants achieve a higher score than the top-ranked benign variant. Abbreviations: LQTS=Long QT syndrome, BrS=Brugada Syndrome, HCM=Hypertrophic Cardiomyopathy, TPR=true positive rate, FPR=false positive rate.

Supplemental tables

Table S1: LQTS training data. Variants are sorted by gene and then by position. 1KG and ESP denote allele frequencies in the 1000 Genome project and Exome Sequencing Project respectively. The ESP frequency is the average of European American and African American population frequencies. The conservation classification is derived from one-to-one orthologues in up to 70 species, as described in the Methods. Abbreviations: PPH2 - PolyPhen2, SIFT - Sorting Intolerant From Tolerant, TM - Transmembrane, T./L./P. - Transmembrane/Linker/Pore, SAD - subunits assembly domain, IQ domain - IQ calmodulin-binding motif (IQ = isoleucine glutamine), cNBD - cyclic nucleotide-binding domain, PAS - Per-Arnt-Sim, DAG1 - dystroglycan 1, PH1 - pleckstrin homology domain 1 , non-cons. - non-conserved, path. - pathogenic.

**Variant**	**Gene**	**Domain**	**Variant class**	**1KG**	**ESP**	**PPH2**	**SIFT**	**Grant-ham**	**Conser-vation**	**Disease status**
c.332A>G	KCNQ1	N-terminus	missense	NA	NA	0.997	0.00	194	primates	path.
c.341T>C	KCNQ1	N-terminus	missense	NA	NA	0.954	0.00	98	primates	path.
c.350C>T	KCNQ1	N-terminus	missense	NA	NA	0.918	0.00	98	primates	path.
c.356G>A	KCNQ1	N-terminus	missense	NA	NA	0.271	0.00	94	primates	benign
c.385G>A	KCNQ1	T./L./P. TM helical S1	missense	NA	NA	0.277	0.09	29	primates	benign
c.674C>T	KCNQ1	T./L./P. TM extracellular	missense	NA	NA	0.856	0.02	145	all species	path.
c.889G>A	KCNQ1	T./L./P. TM extracellular	missense	NA	0.00012	0.020	1.00	56	mammals	benign
c.451_452delCT	KCNQ1	T./L./P. TM helical S2	radical	NA	NA	NA	NA	NA	non-cons.	path.
c.513C>G	KCNQ1	T./L./P. TM cytoplasmic	radical	NA	NA	NA	NA	NA	vertebrates	path.
c.520C>T	KCNQ1	T./L./P. TM cytoplasmic	missense	NA	NA	1.000	0.00	180	all species	path.
c.532G>C	KCNQ1	T./L./P. TM cytoplasmic	missense	NA	NA	0.999	0.00	27	mammals	path.
c.565G>C	KCNQ1	T./L./P. TM cytoplasmic	missense	NA	NA	1.000	0.00	125	all species	path.
c.565G>A	KCNQ1	T./L./P. TM cytoplasmic	missense	NA	NA	1.000	0.00	125	all species	path.
c.569G>A	KCNQ1	T./L./P. TM cytoplasmic	missense	NA	NA	0.999	0.00	43	all species	path.
c.572T>C	KCNQ1	T./L./P. TM cytoplasmic	missense	NA	NA	0.999	0.00	98	mammals	path.
c.572_576 delTGCGC	KCNQ1	T./L./P. TM cytoplasmic	radical	NA	NA	NA	NA	NA	non-cons.	path.
c.573_577 delGCGCT	KCNQ1	T./L./P. TM cytoplasmic	radical	NA	NA	NA	NA	NA	non-cons.	path.
c.585delG	KCNQ1	T./L./P. TM cytoplasmic	radical	NA	NA	NA	NA	NA	non-cons.	path.
c.760G>C	KCNQ1	T./L./P. TM cytoplasmic	missense	NA	NA	0.980	0.00	32	all species	path.
c.760G>T	KCNQ1	T./L./P. TM cytoplasmic	missense	NA	NA	0.980	0.00	32	all species	path.
c.760G>A	KCNQ1	T./L./P. TM cytoplasmic	missense	NA	NA	0.996	0.00	21	all species	path.
c.773A>G	KCNQ1	T./L./P. TM cytoplasmic	missense	NA	NA	0.889	0.00	29	mammals	path.
c.775C>T	KCNQ1	T./L./P. TM cytoplasmic	missense	NA	NA	0.992	0.00	180	primates	path.
c.783G>C	KCNQ1	T./L./P. TM cytoplasmic	missense	NA	NA	0.993	0.00	45	all species	path.
c.541C>T	KCNQ1	T./L./P. TM cytoplasmic	missense	NA	NA	0.402	0.40	180	vertebrates	benign
c.584G>A	KCNQ1	T./L./P. TM cytoplasmic	missense	NA	0.00017	0.957	0.01	43	primates	benign
c.604G>A	KCNQ1	T./L./P. TM helical S3	missense	NA	NA	0.999	0.00	23	all species	path.
c.604G>C	KCNQ1	T./L./P. TM helical S3	missense	NA	NA	1.000	0.00	81	all species	path.
c.610A>T	KCNQ1	T./L./P. TM helical S3	missense	NA	NA	0.708	0.04	21	mammals	path.
c.612C>G	KCNQ1	T./L./P. TM helical S3	missense	NA	NA	0.926	0.29	10	mammals	path.
c.626C>T	KCNQ1	T./L./P. TM helical S3	missense	NA	NA	0.932	0.02	155	all species	path.
c.643G>A	KCNQ1	T./L./P. TM helical S3	missense	NA	NA	0.756	0.21	21	primates	path.
c.691C>T	KCNQ1	T./L./P. TM helical S4 voltage sensor	missense	NA	NA	1.000	0.00	180	primates	path.
c.704T>A	KCNQ1	T./L./P. TM helical S4 voltage sensor	missense	NA	NA	0.922	0.00	149	mammals	path.
c.716T>C	KCNQ1	T./L./P. TM helical S4 voltage sensor	missense	NA	NA	0.996	0.00	98	mammals	path.
c.727C>T	KCNQ1	T./L./P. TM helical S4 voltage sensor	missense	NA	NA	0.975	0.00	180	mammals	path.
c.728G>A	KCNQ1	T./L./P. TM helical S4 voltage sensor	missense	NA	NA	0.998	0.00	29	mammals	path.
c.805G>A	KCNQ1	T./L./P. TM helical S5	missense	NA	NA	0.965	0.00	56	all species	path.
c.806G>A	KCNQ1	T./L./P. TM helical S5	missense	NA	NA	0.998	0.00	94	all species	path.
c.815G>T	KCNQ1	T./L./P. TM helical S5	missense	NA	NA	0.240	0.28	109	mammals	path.
c.817C>T	KCNQ1	T./L./P. TM helical S5	missense	NA	NA	0.992	0.00	22	all species	path.
c.824T>C	KCNQ1	T./L./P. TM helical S5	missense	NA	NA	0.867	0.00	155	all species	path.
c.830C>T	KCNQ1	T./L./P. TM helical S5	missense	NA	NA	0.997	0.00	145	all species	path.
c.916G>A	KCNQ1	T./L./P. TM pore-forming H5	missense	NA	NA	0.989	0.00	125	all species	path.
c.916G>C	KCNQ1	T./L./P. TM pore-forming H5	missense	NA	NA	0.989	0.00	125	all species	path.
c.919G>C	KCNQ1	T./L./P. TM pore-forming H5	missense	NA	NA	0.588	0.36	32	all species	path.
c.935C>T	KCNQ1	T./L./P. TM pore-forming H5	missense	NA	NA	1.000	0.00	89	mammals	path.
c.940G>A	KCNQ1	T./L./P. TM pore-forming H5	missense	NA	NA	1.000	0.00	56	mammals	path.

continued on the next page...

Table S1 – continued

Variant	Gene	Domain	Variant class	1KG	ESP	PPH2	SIFT	Grant-ham	Conser-vation	Disease status
c.944A>G	KCNQ1	T./L./P. TM pore-forming H5	missense	NA	NA	1.000	0.00	194	primates	path.
c.944A>C	KCNQ1	T./L./P. TM pore-forming H5	missense	NA	NA	1.000	0.00	144	primates	path.
c.958C>G	KCNQ1	T./L./P. TM pore-forming H5	missense	NA	NA	1.000	0.02	27	mammals	path.
c.1017_1019 delCTT	KCNQ1	T./L./P. TM helical S6	inframe	NA	NA	NA	NA	NA	non-cons.	path.
c.1022C>A	KCNQ1	T./L./P. TM helical S6	missense	NA	NA	0.999	0.00	107	all species	path.
c.1027C>T	KCNQ1	T./L./P. TM helical S6	missense	NA	NA	0.998	0.00	74	all species	path.
c.1032G>A	KCNQ1	T./L./P. TM helical S6	radical	NA	NA	NA	NA	NA	non-cons.	path.
c.1034G>A	KCNQ1	T./L./P. TM helical S6	missense	NA	NA	1.000	0.00	98	primates	path.
c.1003T>C	KCNQ1	T./L./P. TM helical S6	missense	NA	NA	0.217	0.86	22	all species	benign
c.1046C>G	KCNQ1	C-terminus	missense	NA	NA	1.000	0.00	177	primates	path.
c.1070A>G	KCNQ1	C-terminus	missense	NA	NA	0.309	0.00	43	primates	path.
c.1097G>A	KCNQ1	C-terminus	missense	NA	NA	0.981	0.00	43	primates	path.
c.1201_1202insC	KCNQ1	C-terminus	radical	NA	NA	NA	NA	NA	non-cons.	path.
c.1552C>T	KCNQ1	C-terminus	radical	NA	0.00006	NA	NA	NA	mammals	path.
c.1588C>T	KCNQ1	C-terminus	radical	NA	NA	NA	NA	NA	all species	path.
c.1615C>T	KCNQ1	C-terminus	missense	NA	NA	0.998	0.00	101	all species	path.
c.1663C>T	KCNQ1	C-terminus	missense	NA	NA	1.000	0.00	180	all species	path.
c.1714delC	KCNQ1	C-terminus	radical	NA	NA	NA	NA	NA	non-cons.	path.
c.1760C>T	KCNQ1	C-terminus	missense	NA	NA	0.846	0.00	81	primates	path.
c.1893delC	KCNQ1	C-terminus	radical	NA	NA	NA	NA	NA	non-cons.	path.
c.1909delC	KCNQ1	C-terminus	radical	NA	NA	NA	NA	NA	non-cons.	path.
c.1283A>G	KCNQ1	C-terminus	missense	NA	NA	0.009	0.21	94	primates	benign
c.1321C>T	KCNQ1	C-terminus	missense	NA	0.00006	0.576	0.07	74	vertebrates	benign
c.1355G>A	KCNQ1	C-terminus	missense	NA	0.00017	0.253	0.54	43	primates	benign
c.1442G>T	KCNQ1	C-terminus	missense	NA	NA	0.374	0.05	97	mammals	benign
c.1451G>C	KCNQ1	C-terminus	missense	NA	NA	0.950	0.46	58	mammals	benign
c.1556G>A	KCNQ1	C-terminus	missense	NA	NA	0.985	0.12	29	mammals	benign
c.1861G>A	KCNQ1	C-terminus	missense	NA	NA	0.002	1.00	56	non-cons.	benign
c.1942G>A	KCNQ1	C-terminus	missense	0.00504	0.00899	0.000	0.20	29	non-cons.	benign
c.1766G>A	KCNQ1	C-terminus SAD	missense	NA	NA	0.969	0.06	94	all species	path.
c.1768G>A	KCNQ1	C-terminus SAD	missense	NA	NA	0.418	0.00	58	mammals	path.
c.1772G>A	KCNQ1	C-terminus SAD	missense	NA	NA	0.993	0.00	29	all species	path.
c.1781G>A	KCNQ1	C-terminus SAD	missense	NA	NA	0.970	0.00	43	all species	path.
c.1831G>T	KCNQ1	C-terminus SAD	missense	NA	NA	0.231	0.09	160	primates	path.
c.82A>G	KCNH2	N-terminus	missense	NA	NA	0.662	0.01	56	primates	path.
c.87C>A	KCNH2	N-terminus	missense	NA	NA	0.025	0.00	22	primates	path.
c.92T>G	KCNH2	N-terminus	missense	NA	NA	0.914	0.00	142	primates	path.
c.232G>C	KCNH2	N-terminus	missense	NA	NA	0.124	0.07	27	primates	path.
c.257T>G	KCNH2	N-terminus	missense	NA	NA	0.033	0.00	102	primates	path.
c.542G>A	KCNH2	N-terminus	missense	0.00504	NA	0.394	0.38	43	non-cons.	benign
c.559_567 delGGCG CGGGC	KCNH2	N-terminus	inframe	NA	NA	NA	NA	NA	non-cons.	benign
c.559G>A	KCNH2	N-terminus	missense	NA	NA	0.002	0.85	56	non-cons.	benign
c.568G>A	KCNH2	N-terminus	missense	NA	NA	0.025	0.73	58	non-cons.	benign
c.607G>A	KCNH2	N-terminus	missense	NA	NA	0.001	0.59	58	non-cons.	benign
c.644T>G	KCNH2	N-terminus	missense	NA	NA	0.244	0.04	109	non-cons.	benign
c.707G>T	KCNH2	N-terminus	missense	NA	NA	0.032	0.27	109	primates	benign
c.751C>G	KCNH2	N-terminus	missense	NA	NA	0.068	0.65	27	mammals	benign
c.762C>G	KCNH2	N-terminus	missense	NA	NA	0.002	0.38	24	primates	benign
c.769A>C	KCNH2	N-terminus	missense	NA	NA	0.006	0.52	68	mammals	benign
c.1099A>T	KCNH2	N-terminus	missense	NA	NA	0.235	0.15	58	primates	benign
c.127T>G	KCNH2	N-terminus Per-Arnt-Sim	missense	NA	NA	0.491	0.00	160	primates	path.
c.128A>G	KCNH2	N-terminus Per-Arnt-Sim	missense	NA	NA	0.021	0.00	194	primates	path.
c.157G>C	KCNH2	N-terminus Per-Arnt-Sim	missense	NA	NA	0.990	0.00	125	primates	path.
c.167G>A	KCNH2	N-terminus Per-Arnt-Sim	missense	NA	NA	0.725	0.00	43	primates	path.
c.193A>C	KCNH2	N-terminus Per-Arnt-Sim	missense	NA	NA	0.994	0.01	38	all species	path.
c.196T>G	KCNH2	N-terminus Per-Arnt-Sim	missense	NA	NA	1.000	0.00	159	all species	path.
c.209A>G	KCNH2	N-terminus Per-Arnt-Sim	missense	NA	NA	0.941	0.29	29	primates	path.
c.332A>T	KCNH2	N-terminus PAS-associated C terminal	missense	NA	NA	0.430	0.16	152	primates	path.
c.371T>G	KCNH2	N-terminus PAS-associated C terminal	missense	NA	NA	0.042	0.04	91	primates	path.
c.371T>C	KCNH2	N-terminus PAS-associated C terminal	missense	NA	NA	0.854	0.00	81	primates	path.
c.1262C>T	KCNH2	T./L./P. TM helical S1	missense	NA	NA	0.993	0.00	81	all species	path.
c.1264G>A	KCNH2	T./L./P. TM helical S1	missense	NA	NA	0.979	0.00	58	all species	path.
c.1307C>T	KCNH2	T./L./P. TM extracellular	missense	NA	NA	0.018	0.16	81	primates	path.

continued on the next page. . .

Table S1 – continued

Variant	Gene	Domain	Variant class	1KG	ESP	PPH2	SIFT	Grant-ham	Conser-vation	Disease status
c.1711A>C	KCNH2	T./L./P. TM extracellular	missense	NA	NA	0.974	0.00	5	all species	path.
c.1714G>A	KCNH2	T./L./P. TM extracellular	missense	NA	NA	0.985	0.00	56	all species	path.
c.1714G>C	KCNH2	T./L./P. TM extracellular	missense	NA	NA	0.999	0.00	125	all species	path.
c.1744C>T	KCNH2	T./L./P. TM extracellular	missense	NA	NA	0.964	0.00	180	non-cons.	path.
c.1750G>A	KCNH2	T./L./P. TM extracellular	missense	NA	NA	0.784	0.16	56	all species	path.
c.1778T>G	KCNH2	T./L./P. TM extracellular	missense	NA	NA	0.926	0.00	97	mammals	path.
c.1783A>G	KCNH2	T./L./P. TM extracellular	missense	NA	NA	0.262	0.10	56	all species	path.
c.1787C>G	KCNH2	T./L./P. TM extracellular	missense	NA	NA	0.102	0.74	103	mammals	path.
c.1801G>A	KCNH2	T./L./P. TM extracellular	missense	NA	NA	0.058	0.47	56	mammals	path.
c.1810G>A	KCNH2	T./L./P. TM extracellular	missense	NA	NA	0.995	0.00	56	all species	path.
c.1831T>C	KCNH2	T./L./P. TM extracellular	missense	NA	NA	0.980	0.00	83	all species	path.
c.1897A>G	KCNH2	T./L./P. TM extracellular	missense	NA	NA	0.823	0.01	23	all species	path.
c.1898A>G	KCNH2	T./L./P. TM extracellular	missense	NA	NA	0.801	0.01	46	all species	path.
c.1366G>T	KCNH2	T./L./P. TM helical S2	missense	NA	NA	0.927	0.00	160	all species	path.
c.1387T>C	KCNH2	T./L./P. TM helical S2	missense	NA	NA	0.919	0.00	22	all species	path.
c.1408A>G	KCNH2	T./L./P. TM helical S2	missense	NA	NA	0.992	0.00	23	all species	path.
c.1421C>T	KCNH2	T./L./P. TM cytoplasmic	missense	NA	NA	0.987	0.00	89	all species	path.
c.1468G>A	KCNH2	T./L./P. TM cytoplasmic	missense	NA	NA	0.947	0.00	58	all species	path.
c.1474C>T	KCNH2	T./L./P. TM cytoplasmic	missense	NA	NA	0.986	0.00	83	primates	path.
c.1539C>A	KCNH2	T./L./P. TM helical S3	missense	NA	NA	0.262	0.03	22	non-cons.	benign
c.1539C>G	KCNH2	T./L./P. TM helical S3	missense	NA	NA	0.262	0.03	22	non-cons.	benign
c.1600C>T	KCNH2	T./L./P. TM helical S4 voltage sensor	missense	NA	NA	1.000	0.00	180	all species	path.
c.1681G>A	KCNH2	T./L./P. TM helical S5	missense	NA	NA	0.981	0.00	58	all species	path.
c.1682C>T	KCNH2	T./L./P. TM helical S5	missense	NA	NA	0.981	0.00	64	all species	path.
c.1685A>C	KCNH2	T./L./P. TM helical S5	missense	NA	NA	0.722	0.00	77	all species	path.
c.1834G>T	KCNH2	T./L./P. TM pore-forming H5	missense	NA	NA	0.267	0.07	32	all species	path.
c.1841C>T	KCNH2	T./L./P. TM pore-forming H5	missense	NA	NA	0.990	0.00	64	all species	path.
c.1868C>T	KCNH2	T./L./P. TM pore-forming H5	missense	NA	NA	0.979	0.00	89	all species	path.
c.1882G>A	KCNH2	T./L./P. TM pore-forming H5	missense	NA	NA	0.997	0.00	56	all species	path.
c.1885A>G	KCNH2	T./L./P. TM pore-forming H5	missense	NA	NA	0.996	0.00	23	all species	path.
c.1886A>G	KCNH2	T./L./P. TM pore-forming H5	missense	NA	NA	0.951	0.00	46	all species	path.
c.1888G>C	KCNH2	T./L./P. TM pore-forming H5	missense	NA	NA	0.797	0.00	32	all species	path.
c.1889T>C	KCNH2	T./L./P. TM pore-forming H5	missense	NA	NA	0.963	0.00	64	all species	path.
c.1891T>G	KCNH2	T./L./P. TM pore-forming H5	missense	NA	NA	0.557	0.27	99	all species	path.
c.1918T>G	KCNH2	T./L./P. TM helical S6	missense	NA	NA	0.991	0.00	50	all species	path.
c.2086C>T	KCNH2	C-terminus	missense	NA	NA	0.999	0.00	180	all species	path.
c.2092G>T	KCNH2	C-terminus	radical	NA	NA	NA	NA	NA	all species	path.
c.2536C>A	KCNH2	C-terminus	missense	NA	NA	0.987	0.06	38	all species	path.
c.2587C>T	KCNH2	C-terminus	radical	NA	NA	NA	NA	NA	all species	path.
c.2592+1G>A	KCNH2	C-terminus	radical	NA	NA	NA	NA	NA	non-cons.	path.
c.2764C>T	KCNH2	C-terminus	missense	NA	NA	0.898	0.02	101	primates	path.
c.2768delC	KCNH2	C-terminus	radical	NA	NA	NA	NA	NA	non-cons.	path.
c.2775_2776insG	KCNH2	C-terminus	radical	NA	NA	NA	NA	NA	non-cons.	path.
c.2781G>A	KCNH2	C-terminus	radical	NA	NA	NA	NA	NA	mammals	path.
c.3002G>A	KCNH2	C-terminus	radical	NA	NA	NA	NA	NA	all species	path.
c.3003G>A	KCNH2	C-terminus	radical	NA	NA	NA	NA	NA	all species	path.
c.3040C>T	KCNH2	C-terminus	radical	NA	NA	NA	NA	NA	mammals	path.
c.3255_3256insG	KCNH2	C-terminus	radical	NA	NA	NA	NA	NA	non-cons.	path.
c.2624C>T	KCNH2	C-terminus	missense	NA	NA	0.112	0.08	81	non-cons.	benign
c.2690A>G	KCNH2	C-terminus	missense	NA	0.13886	0.000	0.66	26	primates	benign
c.2690A>T	KCNH2	C-terminus	missense	NA	0.86113	0.063	0.02	95	primates	benign
c.2729C>T	KCNH2	C-terminus	missense	NA	NA	0.001	0.43	98	primates	benign
c.2731_2778del	KCNH2	C-terminus	inframe	NA	NA	NA	NA	NA	non-cons.	benign
c.2744C>T	KCNH2	C-terminus	missense	NA	NA	0.011	0.31	64	primates	benign
c.2893G>A	KCNH2	C-terminus	missense	NA	NA	0.001	0.56	125	primates	benign
c.2900C>T	KCNH2	C-terminus	missense	NA	NA	0.000	0.15	98	non-cons.	benign
c.2932G>A	KCNH2	C-terminus	missense	NA	NA	0.006	0.58	56	primates	benign
c.2941A>G	KCNH2	C-terminus	missense	0.00046	NA	0.002	0.41	56	mammals	benign
c.3046C>T	KCNH2	C-terminus	missense	NA	NA	0.015	0.70	74	mammals	benign
c.3047C>T	KCNH2	C-terminus	missense	NA	NA	0.142	0.19	98	mammals	benign
c.3058C>T	KCNH2	C-terminus	missense	NA	NA	0.007	0.83	74	primates	benign
c.3067_3069del	KCNH2	C-terminus	inframe	NA	NA	NA	NA	NA	non-cons.	benign
c.3077C>T	KCNH2	C-terminus	missense	NA	NA	0.497	0.69	98	mammals	benign
c.3103C>T	KCNH2	C-terminus	missense	NA	NA	0.877	0.18	101	mammals	benign
c.3109G>A	KCNH2	C-terminus	missense	NA	NA	0.128	0.07	23	mammals	benign
c.3164G>A	KCNH2	C-terminus	missense	0.00046	NA	0.022	0.40	43	all species	benign
c.3173C>A	KCNH2	C-terminus	missense	NA	NA	0.032	0.13	107	primates	benign
c.3203A>G	KCNH2	C-terminus	missense	NA	NA	0.242	0.03	43	all species	benign

continued on the next page. . .

Table S1 – continued

Variant	Gene	Domain	Variant class	1KG	ESP	PPH2	SIFT	Grant-ham	Conser-vation	Disease status
c.3289G>A	KCNH2	C-terminus	missense	NA	NA	0.000	0.17	29	primates	benign
c.3322C>G	KCNH2	C-terminus	missense	NA	NA	0.534	0.48	32	mammals	benign
c.3331-7 delAinsGT	KCNH2	C-terminus	radical	0.01282	NA	NA	NA	NA	non-cons.	benign
c.3331-9 _3331-8 delGT	KCNH2	C-terminus	radical	0.01282	NA	NA	NA	NA	non-cons.	benign
c.3331-7delA	KCNH2	C-terminus	radical	0.01282	NA	NA	NA	NA	non-cons.	benign
c.3355G>C	KCNH2	C-terminus	missense	NA	NA	0.968	0.51	29	mammals	benign
c.3460G>A	KCNH2	C-terminus	missense	NA	NA	0.072	0.53	56	mammals	benign
c.2254C>T	KCNH2	C-terminus cNBD	missense	NA	NA	0.999	0.00	101	all species	path.
c.2414T>G	KCNH2	C-terminus cNBD	missense	NA	NA	0.996	0.00	205	all species	path.
c.2453C>T	KCNH2	C-terminus cNBD	missense	NA	NA	0.979	0.00	145	all species	path.
c.2464G>A	KCNH2	C-terminus cNBD	missense	NA	NA	0.997	0.00	21	all species	path.
c.2467C>T	KCNH2	C-terminus cNBD	missense	NA	NA	0.997	0.00	101	all species	path.
c.101G>A	SCN5A	N-terminus	missense	NA	NA	0.253	0.02	29	mammals	benign
c.856G>C	SCN5A	TM 1 TM pore segment	missense	NA	NA	0.066	0.24	27	non-cons.	benign
c.856G>A	SCN5A	TM 1 TM pore segment	missense	NA	NA	0.023	0.56	58	non-cons.	benign
c.856G>T	SCN5A	TM 1 TM pore segment	missense	0.00092	NA	0.014	0.73	99	non-cons.	benign
c.872A>G	SCN5A	TM 1 TM pore segment	missense	NA	NA	0.192	0.16	46	all species	benign
c.895T>A	SCN5A	TM 1 TM pore segment	missense	NA	0.99911	0.026	0.37	15	non-cons.	benign
c.1126C>T	SCN5A	TM 1 TM pore segment	missense	NA	NA	0.981	0.00	180	mammals	benign
c.1231G>A	SCN5A	TM 1 TM domain S6	missense	NA	NA	0.999	0.00	21	all species	path.
c.1715C>A	SCN5A	Interdomain linker I-II	missense	NA	NA	0.052	0.16	126	primates	path.
c.1340C>G	SCN5A	Interdomain linker I-II	missense	NA	0.99962	0.444	0.25	60	mammals	benign
c.1345A>G	SCN5A	Interdomain linker I-II	missense	NA	NA	0.000	0.68	58	non-cons.	benign
c.1383G>T	SCN5A	Interdomain linker I-II	missense	NA	NA	0.005	0.70	22	primates	benign
c.1425A>C	SCN5A	Interdomain linker I-II	missense	NA	NA	0.895	0.32	110	non-cons.	benign
c.1441C>T	SCN5A	Interdomain linker I-II	missense	0.00321	NA	0.005	0.01	101	primates	benign
c.1703G>A	SCN5A	Interdomain linker I-II	missense	NA	NA	0.003	0.04	29	primates	benign
c.1776C>A	SCN5A	Interdomain linker I-II	missense	NA	NA	0.346	0.26	94	non-cons.	benign
c.1787A>G	SCN5A	Interdomain linker I-II	missense	NA	NA	0.908	0.09	94	mammals	benign
c.1802T>C	SCN5A	Interdomain linker I-II	missense	NA	NA	0.583	0.13	64	mammals	benign
c.1913G>A	SCN5A	Interdomain linker I-II	missense	NA	NA	0.261	0.36	94	primates	benign
c.1967C>T	SCN5A	Interdomain linker I-II	missense	0.00137	NA	0.850	0.00	98	mammals	benign
c.2014G>A	SCN5A	Interdomain linker I-II	missense	NA	NA	0.001	1.00	58	primates	benign
c.2114C>T	SCN5A	Interdomain linker I-II	missense	NA	NA	0.154	0.00	155	primates	benign
c.2770G>A	SCN5A	TM 2 TM domain S6	missense	NA	NA	0.050	0.00	29	primates	benign
c.2821_2822 delTCinsAA	SCN5A	Interdomain linker II-III	inframe	NA	NA	NA	NA	NA	non-cons.	path.
c.2911C>G	SCN5A	Interdomain linker II-III	missense	NA	NA	0.133	0.22	125	non-cons.	benign
c.2957G>A	SCN5A	Interdomain linker II-III	missense	NA	NA	0.002	0.37	43	primates	benign
c.3047C>T	SCN5A	Interdomain linker II-III	missense	NA	NA	0.206	0.22	81	non-cons.	benign
c.3118G>A	SCN5A	Interdomain linker II-III	missense	NA	NA	0.012	0.08	125	non-cons.	benign
c.3183A>T	SCN5A	Interdomain linker II-III	missense	NA	NA	0.011	0.42	45	non-cons.	benign
c.3183A>C	SCN5A	Interdomain linker II-III	missense	NA	NA	0.011	0.42	45	non-cons.	benign
c.3229_3231 delCAG	SCN5A	Interdomain linker II-III	inframe	NA	NA	NA	NA	NA	non-cons.	benign
c.3245T>C	SCN5A	Interdomain linker II-III	missense	NA	NA	0.002	0.63	64	primates	benign
c.3252delC +3263insC	SCN5A	Interdomain linker II-III	inframe	NA	NA	NA	NA	NA	non-cons.	benign
c.3292G>T	SCN5A	Interdomain linker II-III	missense	NA	NA	0.048	0.23	32	primates	benign
c.3308C>A	SCN5A	Interdomain linker II-III	missense	0.01190	NA	0.438	0.00	144	primates	benign
c.3346C>T	SCN5A	Interdomain linker II-III	missense	NA	NA	0.003	0.00	101	non-cons.	benign
c.3751G>A	SCN5A	TM 3 TM domain S2	missense	NA	NA	0.883	0.00	21	all species	benign
c.3974A>G	SCN5A	TM 3 TM intracellular S4-S5	missense	NA	NA	0.804	0.00	46	primates	path.
c.3995C>T	SCN5A	TM 3 TM intracellular S4-S5	missense	NA	NA	0.990	0.00	98	all species	path.
c.3998C>A	SCN5A	TM 3 TM intracellular S4-S5	missense	NA	NA	0.999	0.00	144	primates	path.
c.4222G>A	SCN5A	TM 3 TM pore segment	missense	NA	NA	0.996	0.00	125	all species	path.
c.4299+2T>A	SCN5A	TM 3 TM pore segment	radical	NA	NA	NA	NA	NA	non-cons.	benign
c.4519_4527 delCAGA AGCCC	SCN5A	Interdomain linker III-IV	inframe	NA	NA	NA	NA	NA	non-cons.	path.
c.4786T>A	SCN5A	TM 4 TM domain S3	missense	NA	NA	0.362	0.03	21	all species	path.
c.4850_4852 delTCT	SCN5A	TM 4 TM extracellular S3-S4	inframe	NA	NA	NA	NA	NA	non-cons.	path.
c.4859C>A	SCN5A	TM 4 TM extracellular S3-S4	missense	NA	NA	0.898	0.00	78	mammals	path.
c.4868G>A	SCN5A	TM 4 TM domain S4 voltage sensor	missense	NA	NA	0.363	0.00	43	mammals	path.

continued on the next page...

Table S1 – continued

Variant	Gene	Domain	Variant class	1KG	ESP	PPH2	SIFT	Grant-ham	Conser-vation	Disease status
c.4877G>C	SCN5A	TM 4 TM domain S4 voltage sensor	missense	NA	NA	0.999	0.00	103	mammals	path.
c.4931G>A	SCN5A	TM 4 TM domain S4 voltage sensor	missense	NA	NA	0.841	0.00	29	all species	path.
c.5300A>G	SCN5A	TM 4 TM domain S6	missense	NA	NA	0.961	0.00	194	all species	path.
c.5369A>G	SCN5A	C-terminus	missense	NA	NA	0.998	0.00	94	all species	path.
c.5387_5388 insTGA	SCN5A	C-terminus	inframe	NA	NA	NA	NA	NA	non-cons.	path.
c.5457T>G	SCN5A	C-terminus	missense	NA	NA	0.874	0.03	45	non-cons.	benign
c.5457T>A	SCN5A	C-terminus	missense	NA	NA	0.874	0.03	45	non-cons.	benign
c.5507T>C	SCN5A	C-terminus	missense	0.00183	NA	0.547	0.02	89	all species	benign
c.5885C>T	SCN5A	C-terminus	missense	NA	NA	0.122	0.01	98	non-cons.	benign
c.5904C>G	SCN5A	C-terminus	missense	NA	0.99988	0.007	0.28	10	primates	benign
c.6017C>T	SCN5A	C-terminus	missense	NA	NA	0.000	0.17	98	primates	benign
c.5701G>A	SCN5A	C-terminus IQ domain	missense	NA	NA	1.000	0.00	56	all species	benign
c.5755C>T	SCN5A	C-terminus IQ domain	missense	NA	NA	0.316	0.00	180	all species	benign
c.4216G>T	ANK2		missense	0.00046	0.00029	0.397	0.00	159	mammals	benign
c.4645C>T	ANK2		missense	0.00412	0.00500	0.316	0.02	101	non-cons.	benign
c.5212delC	ANK2		radical	NA	NA	NA	NA	NA	non-cons.	benign
c.6535G>A	ANK2		missense	0.02015	0.02719	0.041	0.43	56	primates	benign
c.6908C>T	ANK2		missense	0.01969	0.02775	0.002	0.31	64	non-cons.	benign
c.7007T>C	ANK2		missense	0.09707	0.15108	0.000	0.78	64	non-cons.	benign
c.7168G>A	ANK2		missense	0.00183	NA	0.033	0.34	58	non-cons.	benign
c.7732T>C	ANK2		missense	0.00092	0.00805	0.002	0.42	83	primates	benign
c.7769C>G	ANK2		missense	0.00137	0.00012	0.436	0.03	112	primates	benign
c.8404C>T	ANK2		missense	0.16804	0.19410	0.001	0.68	74	primates	benign
c.8962G>A	ANK2		missense	0.01099	0.00783	0.000	0.46	58	non-cons.	benign
c.9755T>C	ANK2		missense	0.00321	0.00583	0.922	0.05	89	mammals	benign
c.9801C>A	ANK2		missense	0.01603	0.01848	0.050	0.12	110	non-cons.	benign
c.11366G>C	ANK2		missense	0.00137	0.00341	0.001	0.62	60	non-cons.	benign
c.11626T>C	ANK2		missense	0.00229	0.00142	0.400	0.04	74	non-cons.	benign
c.1360G>A	ANK2	Ankyrin domain ANK 13	missense	NA	NA	1.000	0.00	125	primates	benign
c.2060A>G	ANK2	Ankyrin domain ANK 20	missense	NA	0.00029	0.154	0.04	46	primates	benign
c.2151_2152insG	ANK2	Ankyrin domain ANK 21	radical	NA	NA	NA	NA	NA	non-cons.	benign
c.5371T>C	ANK2	Repeat-rich region	missense	NA	NA	0.001	0.08	74	primates	benign
c.115G>A	KCNE1	N-terminus	missense	NA	NA	0.004	0.77	23	primates	benign
p.E43N	KCNE1	N-terminus	missense	NA	NA	0.728	0.01	42	primates	benign
c.154G>A	KCNE1	TM region	missense	NA	NA	0.936	0.00	125	mammals	path.
c.155G>C	KCNE1	TM region	missense	NA	NA	0.927	0.06	60	mammals	benign
c.221C>T	KCNE1	C-terminus	missense	0.00046	NA	0.992	0.00	145	mammals	path.
c.292C>T	KCNE1	C-terminus	missense	NA	NA	0.990	0.00	101	mammals	path.
c.206A>G	KCNE1	C-terminus	missense	NA	NA	0.095	0.17	26	primates	benign
c.220T>G	KCNE1	C-terminus	missense	NA	NA	0.905	0.01	99	mammals	benign
c.235A>G	KCNE1	C-terminus	missense	NA	NA	0.956	0.05	23	primates	benign
c.23C>T	KCNE2	N-terminus	missense	NA	NA	0.997	0.00	89	mammals	benign
c.197C>T	KCNE2	TM region	missense	NA	NA	0.999	0.01	64	all species	benign
c.202T>G	KCNJ2	N-terminus	missense	NA	NA	0.994	0.00	160	all species	path.
c.220A>G	KCNJ2	N-terminus	missense	NA	NA	0.739	0.00	58	all species	path.
c.224C>T	KCNJ2	N-terminus	missense	NA	NA	1.000	0.00	81	all species	path.
c.232G>T	KCNJ2	N-terminus	missense	NA	NA	1.000	0.00	160	all species	path.
c.13C>G	KCNJ2	N-terminus	missense	NA	NA	0.962	0.00	125	mammals	benign
c.143C>A	CACNA1C	N-terminus	missense	NA	NA	0.998	0.18	126	all species	benign
c.1204G>A	CACNA1C	TM domain 1	missense	NA	NA	0.999	0.00	56	all species	path.
c.1140G>A	CACNA1C	TM domain 1	radical	NA	NA	NA	NA	NA	non-cons.	benign
c.1216G>A	CACNA1C	Interdomain linker I-II	missense	NA	NA	1.000	0.01	125	all species	path.
c.2449C>T	CACNA1C	Interdomain linker II-III	missense	0.00366	0.00271	0.356	0.67	74	mammals	benign
c.5383G>A	CACNA1C	C-terminus	missense	0.01328	0.02202	0.024	0.17	125	primates	benign
c.5665C>T	CACNA1C	C-terminus	missense	0.00504	0.00135	0.001	0.18	180	non-cons.	benign
c.233C>T	CAV3	N-terminus DAG1 interaction	missense	0.00183	0.00426	0.738	0.15	81	mammals	path.
c.542T>C	SCN4B	TM region	missense	0.00229	NA	0.467	0.01	98	primates	benign
c.1301G>A	AKAP9		missense	0.01694	0.01487	0.314	0.83	43	primates	benign
c.1389G>T	AKAP9		missense	0.35989	0.45832	0.003	0.20	10	non-cons.	benign
c.2425A>G	AKAP9		missense	0.00229	0.00369	0.001	1.00	29	non-cons.	benign
c.3193A>G	AKAP9		missense	0.00275	NA	0.004	0.31	56	primates	benign
c.3827G>A	AKAP9		missense	0.00962	0.00602	0.001	0.71	43	non-cons.	benign
c.4003_4004 insAAC	AKAP9		inframe	0.39606	NA	NA	NA	NA	non-cons.	benign

continued on the next page...

Table S1 – continued

**Variant**	**Gene**	**Domain**	**Variant class**	**1KG**	**ESP**	**PPH2**	**SIFT**	**Grant-ham**	**Conser-vation**	**Disease status**
c.4005_4006 insCAA	AKAP9		inframe	NA	NA	NA	NA	NA	non-cons.	benign
c.4006_4007 insAAC	AKAP9		inframe	NA	NA	NA	NA	NA	non-cons.	benign
c.4199T>C	AKAP9		missense	0.04350	0.05396	0.000	0.26	81	non-cons.	benign
c.4841G>C	AKAP9		missense	NA	NA	0.823	0.12	103	non-cons.	benign
c.4841G>A	AKAP9		missense	0.00275	0.01248	0.044	0.76	43	non-cons.	benign
c.6134A>G	AKAP9		missense	0.00275	0.00568	0.986	0.07	46	primates	benign
c.7451A>G	AKAP9		missense	0.06685	0.07887	0.001	0.64	26	non-cons.	benign
c.8375A>G	AKAP9		missense	0.29624	0.33455	0.002	0.48	46	primates	benign
c.8485G>A	AKAP9		missense	0.00366	0.00472	0.288	0.06	56	primates	benign
c.8935C>T	AKAP9		missense	NA	0.99796	0.000	1.00	74	non-cons.	benign
c.9092A>G	AKAP9		missense	0.00504	0.00988	0.066	0.13	43	primates	benign
c.9929G>A	AKAP9		missense	0.00275	0.01072	0.001	0.39	43	non-cons.	benign
c.10331A>G	AKAP9		missense	0.01328	0.01955	0.142	0.02	43	primates	benign
c.10840A>G	AKAP9		missense	0.00962	0.00941	0.001	1.00	21	non-cons.	benign
c.10858A>G	AKAP9		missense	0.00366	0.00568	0.013	0.26	29	primates	benign
c.11225G>C	AKAP9		missense	0.00229	0.00329	0.997	0.00	103	primates	benign
c.11714T>C	AKAP9		missense	0.00183	NA	0.003	0.11	81	non-cons.	benign
c.317G>A	SNTA1	PH 1	missense	0.00595	NA	0.031	0.10	43	primates	benign
c.844C>G	KCNJ5	C-terminus	missense	NA	0.98951	0.000	1.00	29	non-cons.	benign

Table S2: Brugada syndrome training data. Variants are sorted by gene and then by position. 1KG and ESP denote allele frequencies in the 1000 Genome project and Exome Sequencing Project respectively. The ESP frequency is the average of European American and African American population frequencies. The conservation classification is derived from one-to-one orthologues in up to 70 species, as described in the Methods. Abbreviations: PPH2 - PolyPhen2, SIFT - Sorting Intolerant From Tolerant, TM - Transmembrane, IQ domain - IQ calmodulin-binding motif (IQ = isoleucine glutamine), non-cons. - non-conserved, path. - pathogenic.

**Variant**	**Gene**	**Domain**	**Variant class**	**1KG**	**ESP**	**PPH2**	**SIFT**	**Grantham**	**Conservation**	**Disease status**
c.101G>A	SCN5A	N-terminus	missense	NA	NA	0.253	0.02	29	mammals	benign
c.481G>A	SCN5A	TM 1 TM domain S2	missense	NA	0.00006	0.873	0.00	56	mammals	path.
c.560C>T	SCN5A	TM 1 TM intracellular S2-S3	missense	NA	NA	0.958	0.00	89	all species	path.
c.808C>A	SCN5A	TM 1 TM domain S5	missense	NA	NA	0.936	0.00	53	all species	path.
c.845G>A	SCN5A	TM 1 TM pore segment	missense	NA	NA	0.980	0.04	29	mammals	path.
c.856G>A	SCN5A	TM 1 TM pore segment	missense	NA	NA	0.023	0.56	58	non-cons.	benign
c.856G>T	SCN5A	TM 1 TM pore segment	missense	0.00092	0.00115	0.014	0.73	99	non-cons.	benign
c.856G>C	SCN5A	TM 1 TM pore segment	missense	NA	NA	0.066	0.24	27	non-cons.	benign
c.872A>G	SCN5A	TM 1 TM pore segment	missense	NA	NA	0.192	0.16	46	all species	benign
c.895T>A	SCN5A	TM 1 TM pore segment	missense	NA	0.00090	0.026	0.37	15	non-cons.	benign
c.1126C>T	SCN5A	TM 1 TM pore segment	missense	NA	NA	0.981	0.00	180	mammals	benign
c.1651G>A	SCN5A	Interdomain linker I-II	missense	NA	NA	0.516	0.64	58	primates	path.
c.1340C>G	SCN5A	Interdomain linker I-II	missense	NA	0.00038	0.444	0.25	60	mammals	benign
c.1345A>G	SCN5A	Interdomain linker I-II	missense	NA	NA	0.000	0.68	58	non-cons.	benign
c.1383G>T	SCN5A	Interdomain linker I-II	missense	NA	NA	0.005	0.70	22	primates	benign
c.1425A>C	SCN5A	Interdomain linker I-II	missense	NA	0.00026	0.895	0.32	110	non-cons.	benign
c.1441C>T	SCN5A	Interdomain linker I-II	missense	0.00321	0.00496	0.005	0.01	101	primates	benign
c.1703G>A	SCN5A	Interdomain linker I-II	missense	NA	0.00012	0.003	0.04	29	primates	benign
c.1776C>A	SCN5A	Interdomain linker I-II	missense	NA	NA	0.346	0.26	94	non-cons.	benign
c.1787A>G	SCN5A	Interdomain linker I-II	missense	NA	NA	0.908	0.09	94	mammals	benign
c.1802T>C	SCN5A	Interdomain linker I-II	missense	NA	NA	0.583	0.13	64	mammals	benign
c.1913G>A	SCN5A	Interdomain linker I-II	missense	NA	NA	0.261	0.36	94	primates	benign
c.1967C>T	SCN5A	Interdomain linker I-II	missense	0.00137	0.00170	0.850	0.00	98	mammals	benign
c.2014G>A	SCN5A	Interdomain linker I-II	missense	NA	0.00006	0.001	1.00	58	primates	benign
c.2114C>T	SCN5A	Interdomain linker I-II	missense	NA	NA	0.154	0.00	155	primates	benign
c.2465G>A	SCN5A	TM 2 TM domain S4 voltage sensor	radical	NA	NA	NA	NA	NA	primates	path.
c.2770G>A	SCN5A	TM 2 TM domain S6	missense	NA	NA	0.050	0.00	29	primates	benign
c.2632C>T	SCN5A	TM 2 TM pore segment	missense	NA	NA	0.959	0.00	180	all species	path.
c.2893C>T	SCN5A	Interdomain linker II-III	missense	NA	NA	0.989	0.00	180	non-cons.	path.
c.2911C>G	SCN5A	Interdomain linker II-III	missense	NA	NA	0.133	0.22	125	non-cons.	benign
c.2957G>A	SCN5A	Interdomain linker II-III	missense	NA	0.00006	0.002	0.37	43	primates	benign
c.3047C>T	SCN5A	Interdomain linker II-III	missense	NA	NA	0.206	0.22	81	non-cons.	benign
c.3118G>A	SCN5A	Interdomain linker II-III	missense	NA	0.00012	0.012	0.08	125	non-cons.	benign
c.3183A>C	SCN5A	Interdomain linker II-III	missense	NA	NA	0.011	0.42	45	non-cons.	benign
c.3183A>T	SCN5A	Interdomain linker II-III	missense	NA	NA	0.011	0.42	45	non-cons.	benign
c.3229_3231 delCAG	SCN5A	Interdomain linker II-III	inframe	NA	NA	NA	NA	NA	non-cons.	benign
c.3245T>C	SCN5A	Interdomain linker II-III	missense	NA	NA	0.002	0.63	64	primates	benign
c.3252delC +3263insC	SCN5A	Interdomain linker II-III	inframe	NA	NA	NA	NA	NA	non-cons.	benign
c.3292G>T	SCN5A	Interdomain linker II-III	missense	NA	NA	0.048	0.23	32	primates	benign
c.3308C>A	SCN5A	Interdomain linker II-III	missense	0.01190	0.03662	0.438	0.00	144	primates	benign
c.3346C>T	SCN5A	Interdomain linker II-III	missense	NA	NA	0.003	0.00	101	non-cons.	benign
c.3751G>A	SCN5A	TM 3 TM domain S2	missense	NA	0.00108	0.883	0.00	21	all species	benign
c.3840+1G>A	SCN5A	TM 3 TM domain S3	radical	NA	NA	NA	NA	NA	non-cons.	path.
c.4018G>A	SCN5A	TM 3 TM domain S5	missense	NA	NA	0.996	0.00	29	primates	path.
c.3956G>T	SCN5A	TM 3 TM intracellular S4-S5	missense	NA	0.00012	1.000	0.00	109	all species	path.
c.3995C>T	SCN5A	TM 3 TM intracellular S4-S5	missense	NA	NA	0.990	0.00	98	all species	path.
c.4222G>A	SCN5A	TM 3 TM pore segment	missense	NA	NA	0.996	0.00	125	all species	path.
c.4299+2T>A	SCN5A	TM 3 TM pore segment	radical	NA	NA	NA	NA	NA	non-cons.	benign
c.4732_4733 dupAA	SCN5A	TM 4 TM domain S2	radical	NA	NA	NA	NA	NA	non-cons.	path.
c.4850_4852 delTCT	SCN5A	TM 4 TM extracellular S3-S4	inframe	NA	NA	NA	NA	NA	non-cons.	path.
c.4868G>A	SCN5A	TM 4 TM domain S4 voltage sensor	missense	NA	NA	0.363	0.00	43	mammals	path.
c.5457T>A	SCN5A	C-terminus	missense	NA	NA	0.874	0.03	45	non-cons.	benign
c.5457T>G	SCN5A	C-terminus	missense	NA	NA	0.874	0.03	45	non-cons.	benign
c.5507T>C	SCN5A	C-terminus	missense	0.00183	0.00129	0.547	0.02	89	all species	benign
c.5885C>T	SCN5A	C-terminus	missense	NA	0.00018	0.122	0.01	98	non-cons.	benign
c.5904C>G	SCN5A	C-terminus	missense	NA	0.00012	0.007	0.28	10	primates	benign

continued on the next page...

Table S2 – continued

**Variant**	**Gene**	**Domain**	**Variant class**	**1KG**	**ESP**	**PPH2**	**SIFT**	**Grant-ham**	**Conser-vation**	**Disease status**
c.6017C>T	SCN5A	C-terminus	missense	NA	NA	0.000	0.17	98	primates	benign
c.5701G>A	SCN5A	C-terminus IQ domain	missense	NA	0.00017	1.000	0.00	56	all species	benign
c.5755C>T	SCN5A	C-terminus IQ domain	missense	NA	NA	0.316	0.00	180	all species	benign
c.143C>A	CACNA1C	N-terminus	missense	NA	NA	0.998	0.18	126	all species	benign
c.1140G>A	CACNA1C	TM domain 1	radical	NA	NA	NA	NA	NA	non-cons.	benign
c.2449C>T	CACNA1C	Interdomain linker II-III	missense	0.00366	0.00271	0.356	0.67	74	mammals	benign
c.5383G>A	CACNA1C	C-terminus	missense	0.01328	0.02202	0.024	0.17	125	primates	benign
c.5665C>T	CACNA1C	C-terminus	missense	0.00504	0.00135	0.001	0.18	180	non-cons.	benign
c.566C>T	SCN1B	Cytoplasmic region	missense	NA	NA	0.662	0.13	81	non-cons.	benign
c.29T>C	SCN3B		missense	NA	0.00012	0.040	0.06	98	non-cons.	path.
c.290G>A	SCN3B	Extracellular region	missense	NA	NA	0.887	0.73	46	mammals	benign
c.719G>A	CACNA2D1		missense	NA	NA	0.882	0.38	26	all species	benign
c.3021G>C	CACNA2D1		missense	NA	NA	0.007	1.00	45	primates	benign
c.3134A>C	CACNA2D1		missense	0.00321	0.00445	0.013	0.32	126	primates	benign
c.1654C>G	CACNB2		missense	0.00733	0.01053	0.619	0.01	125	mammals	benign
c.1803T>G	CACNB2		missense	0.11081	0.14147	0.021	0.05	45	primates	benign
c.532C>T	GPD1L		missense	NA	NA	0.585	0.07	22	primates	benign

Table S3: HCM training data. Two HCM data sets are included in the table, i.e. both our own compendium of previously reported sequence variants in our genes of interest from HGMD professional version 2011.4, UniProt, dbSNP 135 and published case series Jordan et al. (2011); Kapa et al. (2009); Kapplinger et al. (2009) and the training set previously applied to the PolyPhen-HCM classifier. Variants are sorted by gene and then by position. 1KG and ESP denote allele frequencies in the 1000 Genome project and Exome Sequencing Project respectively. The ESP frequency is the average of European American and African American population frequencies. The conservation classification is derived from one-to-one orthologues in up to 70 species, as described in the Methods. Abbreviations: PPH2 - PolyPhen2, SIFT - Sorting Intolerant From Tolerant, Myosin N - Myosin N-terminal SH3-like domain, IQ motif - IQ calmodulin-binding motif (IQ = isoleucine glutamine), TNC - Troponin C, non-cons. - non-conserved, path. - pathogenic.

Variant	Gene	Domain	Variant class	1KG	ESP	PPH2	SIFT	Grantham	Conservation	Disease status
c.2609G>A	MYH7		missense	NA	NA	0.861	0.00	29	all species	path.
c.2722C>G	MYH7		missense	NA	NA	0.997	0.00	32	all species	path.
c.2725A>G	MYH7		missense	NA	NA	0.982	0.07	29	all species	benign
c.3337-3dupC	MYH7		radical	NA	NA	NA	NA	NA	non-cons.	benign
c.3382G>A	MYH7		missense	NA	NA	0.713	0.24	58	mammals	benign
c.4066G>A	MYH7		missense	NA	NA	0.641	0.00	56	all species	path.
c.4498C>T	MYH7		missense	NA	NA	1.000	0.00	101	all species	path.
c.4499G>C	MYH7		missense	NA	NA	1.000	0.00	103	all species	path.
c.4772T>A	MYH7		missense	NA	NA	0.007	1.00	113	all species	benign
c.5117T>C	MYH7		missense	NA	NA	1.000	0.00	98	all species	path.
c.5378T>C	MYH7		missense	NA	NA	1.000	0.00	98	all species	path.
c.5533C>T	MYH7		missense	NA	NA	1.000	0.00	101	all species	path.
c.5647G>A	MYH7		missense	NA	NA	0.806	0.00	56	all species	path.
c.5702A>T	MYH7		missense	NA	NA	0.999	0.01	99	all species	path.
p.K1919N	MYH7		missense	NA	NA	0.999	0.00	94	all species	benign
c.2717A>G	MYH7		missense	NA	NA	0.375	0.01	94	all species	path.
c.2770G>A	MYH7		missense	NA	NA	0.995	0.00	56	all species	path.
c.2788G>A	MYH7		missense	NA	NA	0.995	0.00	56	all species	path.
c.2555T>C	MYH7		missense	NA	NA	0.979	0.00	81	all species	path.
c.2302G>C	MYH7		missense	NA	NA	0.995	0.02	125	all species	path.
c.2633T>C	MYH7		missense	NA	NA	0.992	0.17	64	all species	path.
c.2572C>T	MYH7		missense	NA	NA	0.677	0.00	180	primates	benign
c.2606G>A	MYH7		missense	NA	NA	0.999	0.05	29	all species	benign
c.2658C>G	MYH7		missense	NA	NA	0.624	0.07	45	all species	benign
c.3010C>G	MYH7		missense	NA	NA	0.983	0.00	29	all species	benign
c.2787G>C	MYH7		missense	NA	NA	0.992	0.00	45	all species	benign
c.3137T>G	MYH7		missense	NA	NA	0.991	0.01	91	all species	benign
c.2765T>C	MYH7		missense	NA	NA	0.062	0.32	81	primates	benign
c.92T>C	MYH7		missense	NA	NA	0.300	0.05	22	all species	benign
c.2890G>C	MYH7		missense	NA	0.00035	0.992	0.05	32	all species	benign
c.77C>T	MYH7		missense	0.0032	0.00006	0.015	0.30	64	primates	benign
c.154G>A	MYH7	Myosin N	missense	NA	NA	0.010	0.09	21	non-cons.	benign
c.371C>T	MYH7	Myosin head	missense	NA	NA	0.926	0.00	89	non-cons.	path.
c.485A>G	MYH7	Myosin head	missense	NA	NA	0.996	0.00	194	all species	path.
c.732C>T	MYH7	Myosin head	radical	0.2257	0.34649	NA	NA	NA	non-cons.	benign
c.746G>A	MYH7	Myosin head	missense	NA	NA	0.931	0.00	43	all species	path.
c.767G>A	MYH7	Myosin head	missense	NA	NA	0.998	0.00	98	all species	path.
c.1002C>T	MYH7	Myosin head	radical	0.0105	0.02355	NA	NA	NA	non-cons.	benign
c.1177G>A	MYH7	Myosin head	missense	NA	NA	0.782	0.00	58	all species	benign
c.1207C>T	MYH7	Myosin head	missense	NA	NA	1.000	0.00	101	all species	path.
c.1208G>A	MYH7	Myosin head	missense	NA	NA	0.999	0.00	43	all species	path.
c.1357C>T	MYH7	Myosin head	missense	NA	NA	1.000	0.00	180	all species	path.
c.1594T>C	MYH7	Myosin head	missense	NA	NA	0.888	0.00	74	non-cons.	path.
c.1816G>A	MYH7	Myosin head	missense	NA	NA	0.854	0.00	21	non-cons.	path.
c.2155C>T	MYH7	Myosin head	missense	NA	NA	0.846	0.00	101	all species	path.
c.2156G>A	MYH7	Myosin head	missense	NA	NA	0.294	0.02	43	all species	path.
c.2167C>G	MYH7	Myosin head	missense	NA	NA	0.951	0.03	125	mammals	path.
c.2207T>C	MYH7	Myosin head	missense	NA	NA	0.999	0.01	89	mammals	path.
c.2333A>T	MYH7	Myosin head	missense	NA	NA	0.955	0.00	152	all species	path.
c.2333A>G	MYH7	Myosin head	missense	NA	NA	0.973	0.02	94	all species	path.
c.438G>T	MYH7	Myosin head	missense	NA	NA	0.940	0.00	94	primates	path.
c.619A>C	MYH7	Myosin head	missense	NA	NA	0.551	0.09	53	mammals	path.
c.1988G>A	MYH7	Myosin head	missense	NA	NA	0.823	0.10	29	primates	path.
c.2146G>A	MYH7	Myosin head	missense	NA	NA	0.983	0.03	125	all species	path.
c.2167C>T	MYH7	Myosin head	missense	NA	NA	0.995	0.25	180	mammals	path.
c.2221G>T	MYH7	Myosin head	missense	NA	NA	0.992	0.00	184	all species	path.
c.2221G>A	MYH7	Myosin head	missense	NA	NA	0.930	0.00	125	all species	path.

continued on the next page...

Table S3 – continued

Variant	Gene	Domain	Variant class	1KG	ESP	PPH2	SIFT	Grant-ham	Conser-vation	Disease status
c.1954A>G	MYH7	Myosin head	missense	NA	NA	0.820	0.00	125	non-cons.	path.
c.1987C>T	MYH7	Myosin head	missense	NA	NA	0.939	0.02	180	primates	path.
c.1288G>A	MYH7	Myosin head	missense	NA	NA	0.602	0.00	58	non-cons.	benign
c.1699C>T	MYH7	Myosin head	missense	NA	NA	0.946	0.00	180	non-cons.	benign
c.1727A>G	MYH7	Myosin head	missense	NA	NA	1.000	0.00	29	all species	benign
c.958G>A	MYH7	Myosin head	missense	NA	NA	0.968	0.00	21	all species	benign
c.1128C>A	MYH7	Myosin head	missense	NA	NA	0.797	0.04	45	all species	benign
c.1095G>C	MYH7	Myosin head	missense	NA	NA	0.774	0.00	94	all species	benign
c.2292C>G	MYH7	Myosin head Actin binding	missense	NA	NA	0.182	0.04	22	all species	path.
c.1538T>G	MYH7	Myosin head MYH signature	missense	NA	NA	0.979	0.00	205	all species	path.
c.2389G>A	MYH7	IQ motif	missense	NA	0.00012	0.043	0.12	58	non-cons.	path.
c.4135G>A	MYH7	Myosin tail 1	missense	NA	NA	0.955	0.04	58	all species	path.
c.4909G>A	MYH7	Myosin tail 1	missense	NA	0.00063	0.028	0.14	58	primates	benign
c.5287G>A	MYH7	Myosin tail 1	missense	NA	NA	0.907	0.05	58	primates	benign
c.3236G>A	MYH7	Myosin tail 1	missense	NA	NA	0.212	0.14	43	mammals	benign
c.4010G>A	MYH7	Myosin tail 1	missense	NA	NA	0.133	0.10	43	mammals	benign
c.4076G>A	MYH7	Myosin tail 1	missense	NA	NA	0.971	0.00	29	all species	benign
c.4145G>A	MYH7	Myosin tail 1	missense	NA	NA	0.999	0.00	43	all species	benign
c.4985G>A	MYH7	Myosin tail 1	missense	NA	NA	0.012	0.13	29	non-cons.	benign
c.5135G>C	MYH7	Myosin tail 1	missense	NA	NA	1.000	0.00	103	all species	benign
c.5639G>A	MYH7	Myosin tail 1	missense	NA	NA	0.066	0.02	29	all species	benign
c.5243G>A	MYH7	Myosin tail 1	missense	NA	0.00006	0.936	0.06	194	primates	benign
c.4744G>A	MYH7	Myosin tail 1	missense	NA	NA	0.723	0.00	56	all species	benign
c.5419G>A	MYH7	Myosin tail 1	missense	NA	NA	1.000	0.00	56	all species	benign
c.5013C>G	MYH7	Myosin tail 1	missense	NA	NA	0.076	0.02	10	all species	benign
c.4377G>T	MYH7	Myosin tail 1	missense	NA	0.00006	0.999	0.00	94	primates	benign
c.5293A>G	MYH7	Myosin tail 1	missense	NA	NA	0.992	0.04	21	all species	benign
c.4472C>G	MYH7	Myosin tail 1	missense	0.0078	0.00723	0.030	0.04	112	all species	benign
c.5507C>T	MYH7	Myosin tail 1	missense	NA	NA	0.487	0.01	145	mammals	benign
c.4052C>T	MYH7	Myosin tail 1	missense	NA	NA	0.006	0.04	81	mammals	benign
c.5671A>G	MYH7	Myosin tail 1	missense	NA	NA	0.002	0.54	58	primates	benign
c.3809T>C	MYH7	Myosin tail 1	missense	NA	NA	0.007	0.33	64	primates	benign
c.5071G>A	MYH7	Myosin tail 1	missense	NA	NA	0.027	0.08	21	mammals	benign
c.3981C>A	MYH7	Myosin tail 1	missense	NA	0.00006	0.624	0.01	94	all species	benign
c.61C>T	TNNI3		missense	NA	NA	0.941	0.00	180	all species	path.
c.244C>A	TNNI3		missense	NA	NA	0.997	0.08	38	mammals	benign
c.253T>A	TNNI3		missense	NA	NA	0.159	0.06	15	mammals	benign
c.257C>A	TNNI3		missense	NA	NA	0.000	0.52	126	primates	benign
c.484C>T	TNNI3		missense	NA	NA	0.872	0.00	101	non-cons.	path.
c.547_549delAAG	TNNI3		inframe	NA	NA	NA	NA	NA	non-cons.	path.
c.575G>A	TNNI3		missense	NA	NA	0.994	0.00	29	primates	path.
c.607G>A	TNNI3		missense	NA	NA	0.998	0.03	56	primates	path.
c.616A>C	TNNI3		missense	NA	NA	0.991	0.00	53	primates	path.
c.433C>G	TNNI3	TNC and Actin binding	missense	NA	NA	0.825	0.01	125	all species	path.
c.433C>T	TNNI3	TNC and Actin binding	missense	NA	NA	0.970	0.00	101	all species	path.
c.434G>A	TNNI3	TNC and Actin binding	missense	NA	NA	0.604	0.00	43	all species	path.
c.174G>T	TNNI3	TNC binding	missense	NA	NA	0.958	0.22	94	all species	benign
c.220C>A	TNNI3	TNC binding	missense	NA	NA	0.534	0.13	110	all species	benign
c.235C>T	TNNI3	TNC binding	missense	NA	NA	0.950	0.00	180	mammals	benign
c.53-11_53-7 delCTTCT	TNNT2		radical	0.4693	NA	NA	NA	NA	non-cons.	benign
c.236T>A	TNNT2		missense	NA	NA	0.996	0.00	149	all species	path.
c.274C>T	TNNT2		missense	NA	NA	0.999	0.00	101	all species	path.
c.275G>T	TNNT2		missense	NA	NA	0.995	0.00	102	all species	path.
c.275G>A	TNNT2		missense	NA	NA	0.988	0.03	43	all species	path.
c.281G>T	TNNT2		missense	NA	NA	1.000	0.00	102	all species	path.
c.311C>T	TNNT2		missense	NA	NA	0.216	0.01	64	non-cons.	path.
c.328T>C	TNNT2		missense	NA	NA	0.999	0.00	22	all species	path.
c.328T>A	TNNT2		missense	NA	NA	0.999	0.00	21	all species	path.
c.388C>T	TNNT2		missense	NA	NA	0.992	0.00	180	mammals	path.
c.391C>T	TNNT2		missense	NA	NA	0.999	0.00	101	mammals	path.
c.400C>G	TNNT2		missense	NA	NA	0.991	0.00	125	mammals	path.
c.421C>T	TNNT2		missense	NA	NA	1.000	0.00	101	all species	path.
c.451C>T	TNNT2		missense	NA	NA	0.988	0.00	180	mammals	path.

continued on the next page. . .

Table S3 – continued

**Variant**	**Gene**	**Domain**	**Variant class**	**1KG**	**ESP**	**PPH2**	**SIFT**	**Grant-ham**	**Conser-vation**	**Disease status**
c.476G>A	TNNT2		missense	NA	NA	0.770	0.06	43	mammals	path.
c.487G>A	TNNT2		missense	NA	NA	0.395	0.01	56	primates	path.
c.487_489delGAG	TNNT2		inframe	NA	NA	NA	NA	NA	non-cons.	path.
c.536C>T	TNNT2		missense	NA	NA	0.993	0.00	155	mammals	path.
c.613C>T	TNNT2		missense	NA	NA	0.999	0.00	101	primates	path.
c.614G>T	TNNT2		missense	NA	NA	0.987	0.00	102	primates	path.
c.629_631delAGA	TNNT2		inframe	NA	NA	NA	NA	NA	non-cons.	path.
c.682C>G	TNNT2		missense	NA	NA	0.103	0.01	29	primates	benign
c.732G>T	TNNT2		missense	NA	0.00012	0.881	0.00	45	primates	path.
c.740A>G	TNNT2		missense	NA	NA	0.967	0.08	26	primates	path.
c.758A>G	TNNT2		missense	0.0549	0.08183	0.017	0.23	26	primates	benign
c.808G>A	TNNT2		missense	NA	NA	0.736	0.00	23	all species	path.
c.817A>G	TNNT2		missense	NA	NA	0.909	0.00	56	mammals	path.
c.832C>T	TNNT2		missense	NA	0.00040	0.917	0.02	180	primates	path.
c.833G>C	TNNT2		missense	NA	NA	0.865	0.07	103	primates	path.
c.440C>T	MYBPC3		missense	NA	NA	0.093	0.28	98	primates	benign
c.472G>A	MYBPC3		missense	0.0462	0.05171	0.450	0.00	21	non-cons.	benign
c.744C>A	MYBPC3		missense	NA	NA	1.000	0.00	45	all species	benign
c.772G>A	MYBPC3		missense	NA	NA	0.178	0.01	56	mammals	path.
c.1000G>A	MYBPC3		missense	NA	NA	0.334	0.00	56	primates	path.
c.1144C>T	MYBPC3		missense	0.0142	0.01578	0.987	0.00	101	mammals	benign
c.1624G>C	MYBPC3		missense	NA	0.00012	0.536	0.20	29	all species	path.
c.1633C>A	MYBPC3		missense	NA	NA	0.986	0.00	15	all species	benign
c.2308G>A	MYBPC3		missense	NA	NA	0.820	0.00	23	primates	path.
c.2373dupG	MYBPC3		radical	NA	NA	NA	NA	NA	non-cons.	path.
c.2410C>A	MYBPC3		missense	NA	NA	0.972	0.04	15	mammals	benign
c.2601C>T	MYBPC3		radical	0.0156	0.02390	NA	NA	NA	non-cons.	benign
c.2864_2865delCT	MYBPC3		radical	NA	NA	NA	NA	NA	non-cons.	path.
c.3004C>T	MYBPC3		missense	0.0027	0.00249	1.000	0.00	101	mammals	benign
c.3142C>T	MYBPC3		missense	NA	0.00006	0.849	0.00	180	primates	benign
c.3288G>C	MYBPC3		missense	NA	NA	0.050	0.24	45	non-cons.	benign
c.3288G>T	MYBPC3		missense	NA	NA	0.050	0.24	45	non-cons.	benign

Table S4: Deriving the prior probability that a rare variant in a gene is pathogenic for an individual with a particular syndrome. Four estimates of the burden of pathogenic variants in cases are included in the table, based on four studies of the yields of diagnostic genetic testing for each gene and syndrome (Beckmann et al. 2013; Ackerman et al. 2011; Hedley et al. 2009; Pinto et al. 2011). Missing values are indicative that not all studies reported on all the genes studied here. Beckman et al reported the proportion of "mutation positive" cases attributable to each gene, whereas the other studies report the proportion of all cases attributable. As approximately 70% of high-confidence cases are genotype positive on diagnostic testing, the estimates reported in (Beckmann et al. 2013) have been multiplied by 0.7 before inclusion in the table. The composite value reported in Table 1 is the mean of the upper limit of the ranges reported here. As data is sparse for those genes not routinely sequenced in clinical diagnostic sequencing, estimates of the contributions of these genes may be influenced by ascertainment bias.

**Syndrome**	**Gene**	**Percentage of cases attributable to gene**			
		**Hedley**	**Ackerman**	**Beckman**	**Pinto**
LQTS	KCNQ1	40-55	30-35	29.4-36.4	
LQTS	KCNH2	35-45	25-40	22.4-31.5	
LQTS	SCN5A	2-8	5-10	5.6-9.1	
LQTS	ANK2	<1			
LQTS	KCNE1	<1			
LQTS	KCNE2	<1			
LQTS	KCNJ2	<1			
LQTS	CACNA1C	<1			
LQTS	CAV3	<1			
LQTS	SCN4B	<0.1			
LQTS	AKAP9	<0.1			
LQTS	SNTA1	<0.1			
LQTS	KNCJ5	<0.1			
LQTS	LQT4-13 combined		<5		
LQTS	all genes		75	70	
BrS	SCN5A		20-30		
HCM	MYBPC3		20-45		20-30
HCM	MYH7		15-20		20-30
HCM	TNNT2		1-7		3-5
HCM	TNNI3		1-7		3-5
HCM	all genes		60		56

Table S5: Sensitivity and positive predictive value of predictions for various gene and syndrome combinations. The models used in predictions are either the full model from Fig. 2 trained on data on a single syndrome or a multivariate model trained either on combined LQTS and BrS data or on combined LQTS and HCM data. The number after "PPV" and "Sensitivity" indicates a probability threshold at which PPV or sensitivity has been estimated (sensitivity is expressed as a percentage of pathogenic variants detected).

**Syndrome**	**Gene**	**Model**	**PPV 0.9**	**Sensitivity 0.9**	**PPV 0.95**	**Sensitivity 0.95**	**PPV 0.99**	**Sensitivity 0.99**
LQTS	KCNQ1	Fig. 2	1	79	1	60	1	36
LQTS	KCNH2	Fig. 2	0.999	82	1	67	1	42
LQTS	SCN5A	Fig. 2	1	39	1	28	1	4
LQTS	LQT1-3 combined	Fig. 2	0.999	76	1	60	1	35
BrS	SCN5A	Fig. 2	0.963	3	0.973	3	0.902	0.5
BrS	SCN5A	multivariate	0.998	23	1	8	1	0
HCM	MYBPC3	multivariate	0	0	0	0	0	0
HCM	MYH7	multivariate	0.994	26	0.998	7	1	0
HCM	TNNT2	multivariate	0.985	83	1	72	1	19
HCM	TNNI3	multivariate	1	34	1	22	1	0

References

Ackerman M, Priori S, Willems S, Berul C, Brugada R, Calkins H, Camm A, Ellinor P, Gollob M, Hamilton R, et al. 2011. HRS/EHRA expert consensus statement on the state of genetic testing for the channelopathies and cardiomyopathies: this document was developed as a partnership between the Heart Rhythm Society (HRS) and the European Heart Rhythm Association (EHRA). Europace 13: 1077–1109.

Beckmann BM, Wilde A, Kääb S. 2013. Clinical utility gene card for: long QT syndrome (types 1-13). Doi: 10.1038/ejhg.2013.28.

Hedley P, Jürgensen P, Schlamowitz S, Wangari R, Moolman-Smook J, Brink P, Kanters J, Corfield V, Christiansen M. 2009. The genetic basis of long QT and short QT syndromes: a mutation update 30: 1486–1511.

Jordan D, Kiezun A, Baxter S, Agarwala V, Green R, Murray M, Pugh T, Lebo M, Rehm H, Funke B, et al. 2011. Development and validation of a computational method for assessment of missense variants in hypertrophic cardiomyopathy. Am J Hum Genet 88: 183–192.

Kapa S, Tester D, Salisbury B, Harris-Kerr C, Pungliya M, Alders M, Wilde A, Ackerman M. 2009. Genetic testing for long QT syndrome: distinguishing pathogenic mutations from benign variants. Circulation 120: 1752–1760.

Kapplinger J, Tester D, Salisbury B, Carr J, Harris-Kerr C, Pollevick G, Wilde A, Ackerman M. 2009. Spectrum and prevalence of mutations from the first 2,500 consecutive unrelated patients referred for the FAMILION long QT syndrome genetic test. Heart Rhythm 6: 1297–1303.

Pinto Y, Wilde A, van Rijsingen I, Christiaans I, Deprez R, Elliott P. 2011. Clinical utility gene card for: hypertrophic cardiomyopathy (type 1-14). Eur J Hum Genet 19(8). Doi: 10.1038/ejhg.2010.243.
